# Are inequities decreasing? Birth registration for children under five in low-income and middle-income countries, 1999–2016

**DOI:** 10.1136/bmjgh-2019-001926

**Published:** 2019-12-16

**Authors:** Amiya Bhatia, Nancy Krieger, Jason Beckfield, Aluisio J D Barros, Cesar Victora

**Affiliations:** 1 Department of Social and Behavioral Sciences, Harvard University T H Chan School of Public Health, Boston, Massachusetts, USA; 2 Department of Global Health and Development, London School of Hygiene and Tropical Medicine, London, UK; 3 Department of Sociology, Harvard University, Cambridge, Massachusetts, USA; 4 International Center for Equity in Health, Universidade Federal de Pelotas, Pelotas, RS, Brazil

**Keywords:** child health, epidemiology, public health, descriptive study

## Abstract

**Introduction:**

Although global birth registration coverage has improved from 58% to 71% among children under five globally, inequities in birth registration coverage by wealth, urban/rural location, maternal education and access to a health facility persist. Few studies examine whether inequities in birth registration in low-income and middle-income countries have changed over time.

**Methods:**

We combined information on caregiver reported birth registration of 1.6 million children in 173 publicly available, nationally representative Demographic Health Surveys and Multiple Indicator Cluster Surveys across 67 low-income and middle-income countries between 1999 and 2016. For each survey, we calculated point estimates and 95% CIs for the percentage of children under 5 years without birth registration on average and stratified by sex, urban/rural location and wealth. For each sociodemographic variable, we estimated absolute measures of inequality. We then examined changes in non-registration and inequities between surveys, and annually.

**Results:**

14 out of 67 countries had achieved complete birth registration. Among the remaining 53 countries, 39 countries successfully decreased the percentage of children without birth registration. However, this reduction occurred alongside statistically significant increases in wealth inequities in 9 countries and statistically significant decreases in 10 countries. At the most recent survey, the percentage of children without birth registration was greater than 50% in 16 out of 67 countries.

**Conclusion:**

Although birth registration improved on average, progress in reducing wealth inequities has been limited. Findings highlight the importance of monitoring changes in inequities to improve birth registration, to monitor Sustainable Development Goal 16.9 and to strengthen Civil Registration and Vital Statistics systems.

**Key questions**

**What is already known?**

- Globally, birth registration coverage has improved from 58% to 71% among children under five; however, an estimated 230 million children do not have identification documents.
- Prior studies have established that there are inequities in birth registration coverage by several sociodemographic factors including wealth, urban/rural location, maternal education and access to a health facility.
- Complete birth registration is target 16.9 of the Sustainable Development Goals (SDGs) and crucial to ensuring that Civil Registration and Vital Statistics systems can provide accurate, timely and reliable information about infant mortality and other health outcomes, and monitor inequities.

**What are the new findings?**

- Out of 67 low-income and middle-income countries, 14 countries had almost complete birth registration. On average, 39 countries reduced the percentage of children without birth registration, and in 14 countries the percentage of children without birth registration increased.
- The reductions in average non-registration were not met with corresponding reductions in wealth and urban/rural inequities.
- There were statistically significant reductions in both non-registration and wealth inequities in 10 countries.

**What do the new findings imply?**

- There are large wealth and urban/rural inequities in birth registration which persisted while average coverage improved.
- Children in wealthier households and urban areas benefitted from on average improvements in birth registration.
- Monitoring the reduction of wealth and urban/rural inequities should be central to monitoring progress on SDG 16.9 on universal access to birth registration and legal identity.

## Introduction

Achieving ‘legal identity for all, including birth registration, by 2030’ is goal 16.9 of the Sustainable Development Goals (SDGs).^1^ The target responds to a status quo where an estimated 230 million children under age five^2^ and 1 billion adults are without identification documents, and Civil Registration and Vital Statistics (CRVS) systems in many low-income and middle-income countries (LMICs) are unable to generate accurate and timely information about birth, death, marriage and other vital events.^3–6^

Compounding this problem of under-registration is a status quo where, although UNICEF estimates of birth registration in the past decade indicate a global increase from 58% to 65%,^2 3^ and most recently to 71%,^7^ little is known about global changes in inequities. Both comparative and country-specific cross-sectional studies point to large inequalities in birth registration by socioeconomic status, maternal education, residential location and access to primary care, indicating that birth registration is often unevenly and unfairly distributed among children and communities.^2 8–11^ Such inequalities are unfair, unjust and avoidable and are therefore inequities.^12–14^ In line with this, a cross sectional analysis of over 4 million children in 94 countries reported significant wealth inequities in birth registration in 74 countries and urban/rural inequities in 60 countries.^10^ However, few studies examine whether these inequities have persisted or been addressed over time.

The importance of birth registration—and eliminating inequities in birth registration—is profound. The United Nations defines civil registration as ‘*the continuous, permanent, compulsory and universal recording of the occurrence and characteristics of vital events*’*.* 15 Civil registration and the generation of vital statistics have long been the foundation of public health systems and central to the exercise of government responsibilities, with implications for how individuals are counted or seen by the state and how they in turn are able to—or are excluded from—making claims on the state.^16 17^ CRVS systems are implicated in good governance and enable governments to benefit from an accurate and timely understanding of who is being born, who is marrying and who is dying and from inclusive population denominators all of which underpin policy development, resource allocation and efforts to monitor and address inequities in health outcomes.^6 18^ In the context of improving child health, women’s health and preventing mortality, vital statistics from civil registration allow reliable and timely estimates of infant and under-five mortality rates, the maternal mortality ratio, life expectancy at birth, the crude death rate and indicators of total fertility rate.^4 6 19 20^ However, when CRVS systems are weak, decision makers have to rely on other statistical estimates that vary according to the methods used to develop them. For example, in many LMICs, there is a reliance on modelled estimates based on survey and census data to measure and monitor infant and child mortality.^21–23^ For the 192 states that have ratified the 1989 Convention on the Rights of the Child, birth registration has also become central to the obligation these states have to fulfilling the rights of children.^24^

In addition to the ways in which population-level inequities in birth registration can hinder efforts to measure and monitor population health, children without birth registration can face a range of exclusions and vulnerabilities. Without proof of legal identity, children are often invisible in the eyes of the state,^2 25^ and depending on a country’s citizenship regime, may also be stateless.^25–27^ The absence of birth registration also makes proof of age and family relationships challenging.^28^ Proof of age is often relied on for vaccination, the measurement of malnutrition,^29^ school enrolment, making adjudications about child marriage and a child’s rights in the context of juvenile justice and child migration.^30 31^ Proof of family relationship is particularly important in the context of orphaning and family separation. Without the proof of identity linked to birth registration, accessing government social transfers, the banking system and voter registration is challenging or impossible.^11^ Research also indicates that birth registration can help protect children from abuse and exploitation^32 33^ and is associated with improved health outcomes, including early childhood growth and development.^34–36^

Accordingly, given the lack of data on trends in inequities in birth registration and the importance of birth registration, this study examines the direction and magnitude of changes in on-average birth registration and wealth, urban/rural and gender-based inequalities between 1999 and 2016 in all LMICs with Demographic Health Surveys (DHS)/Multiple Indicator Cluster Surveys (MICS) data to ask whether national-level improvements in birth registration were concurrent with reductions in inequities.

## Methods

### Data sources and design

We combined publicly available, nationally representative DHS and MICS from 67 countries between 1999 and 2016. Both DHS and MICS use multistage cluster sampling, are designed to produce nationally representative estimates of health outcomes at the national, urban–rural and regional levels^37 38^ and are often used to establish national targets and monitor progress on the SDGs. In many LMICs, the absence of comprehensive and functioning civil registries means that questions about birth registration DHS and MICS represent the only source of information about birth registration. The number of countries with household survey data on birth registration increased from approximately 70 in 2000 to over 120 in 2017.^39^ Using data from DHS and MICS allowed us to construct a dataset with the most data points for each country over time where the outcome measure, covariates and sampling design were comparable within and across countries.

Birth registration questions were included in the DHS from 1999 and in the MICS from 2000. For birth registration, all children under 5 years of age residing in fixed households were sampled—irrespective of whether their biological mother is also resident in the household—and interviews were conducted with the caregiver. Children who had died, were living on the street or in state institutions are not included in these surveys and we therefore expect to underestimate both the average percentage of children under five without birth registration as well as inequalities in registration.

Because of our interest in changes in both birth registration coverage and inequalities, only countries with two or more surveys with data on birth registration were included in the analysis. Surveys had to include relevant covariates (household wealth quintile, urban/rural location and sex of the child) and be designed to produce nationally representative estimates that could be compared over time: surveys were excluded if birth registration questions were posed differently across years or if national boundaries changed.

We used publicly available data from the World Bank to create a list of regions and income-groups by country.^40^ To select the final sample of surveys, we used information on the DHS and MICS websites and the dataset repository in the International Centre for Equity in Health at the Federal University of Pelotas Brazil to identify the 100 of the 218 World Bank economies with publicly available DHS and MICS surveys in September 2018. A total of 68 countries had more than one survey that met the inclusion criteria. To create our final sample, we grouped surveys into four waves based on time intervals, which corresponded to the years of MICS and DHS survey rounds: surveys conducted prior to 2004 (wave 1), between 2004 and 2008 (wave 2), between 2009 and 2012 (wave 3) and after 2013 (wave 4). The majority of countries (n=57) had one survey per wave. For a small sample of countries (n=11) where there were two surveys per wave, we retained one survey per wave ensuring we included the oldest and most recent survey to preserve the longest time interval and otherwise selected the survey with the larger sample size. One country was excluded from the final sample after applying survey waves as it had two surveys conducted between 2009 and 2012 (wave 3). Our final sample included 67 countries and 173 surveys.

### Outcome

The primary outcome was the percentage of children under five (0–59 months) without birth registration (also referred to as non-registration) as reported by the caregiver. This is the inverse of the MICS and DHS definition of birth registration and described children who did not have a birth certificate, whose births were not registered with the ‘civil authorities’ or whose caregivers did not know whether the child’s birth had been registered. The denominator was the number of children under five included in the nationally representative survey sample. We defined complete birth registration as non-registration less than or equal to 5%, indicating that most children in the country had their births registered.

The DHS questions on birth registration were consistent over survey rounds. However, changes were made to the calculation of birth registration across MICS rounds: to allow for comparability, we recalculated birth registration estimates from MICS2 and MICS3 according to the indicator definition in MICS4, and the estimates presented here may differ from estimates included in the MICS2 and MICS3 national reports. Online supplementary table S1 provides a description of survey questions.

10.1136/bmjgh-2019-001926.supp1 Supplementary data

### Covariates

We included three sociodemographic covariates for disaggregation and estimating inequalities. The groups we hypothesised to be the ‘best off’ and have the lowest percentage of children under five without birth registration were selected as the reference category for analysis. Covariates included: sex of the child (boys (referent (ref)), girls), residential location (urban (ref), rural) and wealth which was used in the analysis both as an ordinal variable (quintiles from poorest to richest (ref)) and as a binary variable (quintiles 1 and 2, quintiles 3–5 (ref)). Consistent with the DHS and MICS methodology, wealth quintiles were calculated by the survey programme based on a household asset index constructed using principal components analysis where wealth quintile 1, for example, represented the poorest 20% of the households.^41^ Because relevant assets may vary in urban and rural households, separate principal component analyses were carried out in each area and then combined into a single score using a scaling procedure to allow comparability. This score was then divided into quintiles.^42^

### Statistical analyses

#### Cross-sectional analyses

For each survey, we calculated point estimates and 95% CIs for the percentage of children under five without birth registration on average and stratified by sex, residential location and wealth. We estimated the absolute difference in non-registration coverage among girls compared with boys and among children living in rural compared with urban areas. To estimate wealth inequalities, we calculated the slope index of inequality (SII), which accounts for the distribution of individual children across household wealth quintiles.^43^ Absolute measures of inequality are more easily interpretable and less sensitive to small differences than relative measures of inequality. We conducted tests of statistical significance to examine whether estimates were significantly different from zero, the null value, which represents no inequality.

#### Changes in birth registration

All analyses were country-specific and we estimated change in non-registration on average and stratified by covariates. For the primary analyses, we calculated the difference in non-registration between the first available and most recent survey in each country and divided this by the number of years between surveys to estimate annual change. In addition, we estimated change by survey wave. We considered countries which maintained non-registration coverage below 5% between the first and most recent survey to have achieved complete registration.

#### Changes in gender, urban/rural and wealth inequalities in birth registration

To examine changes in inequalities in non-registration over time, we estimated the absolute change in gender, urban/rural and wealth inequalities between the first and last survey, annually and between survey waves. Differences in estimates of inequality were deemed significant if the CIs between the first and most recent survey were not overlapping.

All estimates were weighted to account for the multistage sampling design. Analyses were conducted in Stata 15.

## Results

### Sample

The final sample comprised of 67 countries and 173 nationally representative surveys which included 1.6 million children under five. Table 1 and online supplementary figure S1 show the distribution of countries and surveys included, and the percentage of children under five without birth registration for the first and most recent survey. The final sample included countries from 6 of the 7 World Bank regions ranging from 3 countries in Middle East and North Africa to 32 countries in Sub-Saharan Africa. Most countries included were low-middle (45%) or low-income countries (40%) and only 15% were upper-middle income countries. Of the 173 surveys, 75 (43%) were DHS and 98 (57%) were MICS. Surveys were conducted between 1999 and 2016: 17% before 2004 (Wave 1), 33% between 2004 and 2008 (Wave 2), 28% between 2009 and 2012 (Wave 3), and the remaining 21% were conducted between 2013 and 2016 (Wave 4).

**Table 1:** Percentage of children under five without birth registration at the first and most recent survey in the sample of countries (n=67) and surveys (n=173) included

Region	Country	World Bank income group*	Surveys included		% children under five without birth registration	
			N	Years and data sources	First survey	Most recent survey
East Asia and Pacific	Cambodia	Low income	3	2005 (DHS), 2010 (DHS), 2014 (DHS)	33.6	26.7
	Indonesia	Lower-middle income	2	2007 (DHS), 2012 (DHS)	49.4	33.4
	Lao	Lower-middle income	3	2000 (MICS), 2006 (MICS), 2011 (MICS)	40.6	25.2
	Mongolia	Lower-middle income	4	2000 (MICS), 2005 (MICS), 2010 (MICS), 2013 (MICS)	2.4	0.7
	Myanmar	Lower-middle income	2	2000 (MICS), 2015 (DHS)	39.4	18.7
	Thailand	Upper-middle income	2	2005 (MICS), 2012 (MICS)	0.6	0.5
	Vietnam	Lower-middle income	4	2000 (MICS), 2006 (MICS), 2010 (MICS), 2013 (MICS)	27.8	3.9
Europe and Central Asia	Albania	Lower-middle income	2	2000 (MICS), 2008 (DHS)	1.2	1.4
	Armenia	Lower-middle income	2	2005 (DHS), 2010 (DHS)	3.6	0.4
	Azerbaijan	Lower-middle income	2	2000 (MICS), 2006 (DHS)	3.2	6.4
	Kazakhstan	Upper-middle income	3	2006 (MICS), 2010 (MICS), 2015 (MICS)	0.8	0.3
	Kyrgyzstan	Lower-middle income	3	2005 (MICS), 2012 (DHS), 2014 (MICS)	5.7	2.3
	Macedonia	Upper-middle income	2	2005 (MICS), 2011 (MICS)	6.2	0.3
	Moldova	Lower-middle income	3	2000 (MICS), 2005 (DHS), 2012 (MICS)	2.1	0.4
	Montenegro	Upper-middle income	2	2005 (MICS), 2013 (MICS)	2.1	0.6
	Serbia	Upper-middle income	3	2005 (MICS), 2010 (MICS), 2014 (MICS)	1.0	0.6
	Tajikistan	Low income	3	2000 (MICS), 2005 (MICS), 2012 (DHS)	25.4	11.6
	Turkmenistan	Upper-middle income	2	2006 (MICS), 2015 (MICS)	4.5	0.4
	Ukraine	Lower-middle income	2	2005 (MICS), 2012 (MICS)	0.2	0.2
	Uzbekistan	Low income	2	2000 (MICS), 2006 (MICS)	0.5	0.1
Latin America and the Caribbean	Belize	Lower-middle income	2	2006 (MICS), 2011 (MICS)	5.6	4.8
	Bolivia	Lower-middle income	2	2000 (MICS), 2008 (DHS)	18.4	24.1
	Dominican Rep	Upper-middle income	3	2000 (MICS), 2007 (DHS), 2014 (MICS)	25.4	12.0
	Guyana	Lower-middle income	4	2000 (MICS), 2006 (MICS), 2009 (DHS), 2014 (MICS)	3.5	11.3
	Haiti	Low income	2	2005 (DHS), 2012 (DHS)	18.9	20.3
	Honduras	Lower-middle income	2	2005 (DHS), 2011 (DHS)	6.5	6.4
	Suriname	Upper-middle income	2	2006 (MICS), 2010 (MICS)	3.4	1.1
Middle East and North Africa	Iraq	Lower-middle income	2	2000 (MICS), 2011 (MICS)	1.9	0.8
	State of Palestine	Lower-middle income	2	2010 (MICS), 2014 (MICS)	0.7	0.7
	Yemen	Lower-middle income	2	2006 (MICS), 2013 (DHS)	77.7	69.3
South Asia	Afghanistan	Low income	2	2010 (MICS), 2015 (DHS)	62.7	57.7
	Bangladesh	Lower-middle income	3	2006 (MICS), 2012 (MICS), 2014 (DHS)	90.2	79.8
	India	Lower-middle income	2	2005 (DHS), 2015 (DHS),	58.9	20.3
	Nepal	Low income	3	2006 (DHS), 2011 (DHS), 2016 (DHS)	65.0	43.8
	Pakistan	Lower-middle income	2	2006 (DHS), 2012 (DHS)	73.4	66.4
Sub-Saharan Africa	Angola	Upper-middle income	2	2001 (MICS), 2015 (DHS)	70.8	75.0
	Benin	Low income	2	2006 (DHS), 2011 (DHS)	46.9	19.8
	Burkina Faso	Low income	2	2006 (MICS), 2010 (DHS)	36.3	23.1
	Burundi	Low income	3	2000 (MICS), 2005 (MICS), 2010 (DHS)	25.1	24.8
	CAR	Low income	3	2000 (MICS), 2006(MICS), 2010 (MICS)	27.5	39.0
	Cameroon	Lower-middle income	4	2000 (MICS), 2006 (MICS), 2011 (DHS), 2014 (MICS)	66.1	33.9
	Chad	Low income	3	2000 (MICS), 2010 (MICS), 2014 (DHS)	75.1	88.0
	Comoros	Low income	2	2000 (MICS), 2012 (DHS)	16.6	12.7
	Congo DR	Low income	4	2001 (MICS), 2007 (DHS), 2010 (MICS), 2013 (DHS)	65.8	75.4
	Cote d’Ivoire	Lower-middle income	3	2000 (MICS), 2006 (MICS), 2011 (DHS)	28.2	35.0
	Gambia	Low income	3	2000 (MICS), 2005 (MICS), 2013 (DHS)	67.8	28.0
	Ghana	Lower-middle income	3	2006 (MICS), 2011 (MICS), 2014 (DHS)	48.6	29.3
	Guinea Bissau	Low income	3	2000 (MICS), 2006 (MICS), 2014 (MICS)	57.9	76.3
	Kenya	Lower-middle income	2	2008 (DHS), 2014 (DHS)	40.0	33.1
	Lesotho	Lower-middle income	2	2009 (DHS), 2014 (DHS)	54.9	56.7
	Liberia	Low income	2	2007 (DHS), 2013 (DHS)	96.4	75.4
	Mali	Low income	2	2006 (DHS), 2012 (DHS)	46.7	15.7
	Mauritania	Low income	2	2007 (MICS), 2011 (MICS)	44.2	41.2
	Mozambique	Low income	2	2008 (MICS), 2011 (DHS)	69.2	52.1
	Namibia	Upper-middle income	2	2006 (DHS), 2013 (DHS)	32.9	12.9
	Niger	Low income	3	2000 (MICS), 2006 (DHS), 2012 (DHS)	54.5	36.1
	Nigeria	Lower-middle income	3	2007 (MICS), 2011 (MICS), 2013 (DHS)	76.7	70.2
	Rwanda	Low income	3	2005 (DHS), 2010 (DHS), 2014 (DHS)	17.6	44.0
	S Tome & Principe	Lower-middle income	3	2000 (MICS), 2008 (DHS), 2014 (MICS)	30.1	4.8
	Senegal	Low income	3	2000 (MICS), 2010 (DHS), 2015 (DHS)	34.0	31.7
	Sierra Leone	Low income	4	2000 (MICS), 2008 (DHS), 2010 (MICS), 2013 (DHS)	53.6	23.3
	Swaziland	Lower-middle income	4	2000 (MICS), 2006 (DHS), 2010 (MICS), 2014 (MICS)	47.1	46.5
	Tanzania	Low income	2	2010 (DHS), 2015 (DHS)	83.7	73.6
	Togo	Low income	3	2000 (MICS), 2006 (MICS), 2010 (MICS)	17.9	22.1
	Uganda	Low income	2	2006 (DHS), 2011 (DHS)	79.0	70.1
	Zambia	Lower-middle income	3	1999 (MICS), 2007 (DHS), 2013 (DHS)	90.4	88.7
	Zimbabwe	Low income	3	2005 (DHS), 2010 (DHS), 2015 (DHS)	26.1	56.5

Table shows the distribution of countries and surveys included, and the percentage of children under five without birth registration for both the first and most recent survey. Birth registration information was first collected between 1999 and 2010 in each country. In 54 out of 67 countries (80%), the first survey was conducted either in the year 2000 or in 2005–2006. The most recent surveys with birth registration were conducted between 2006 and 2016 and in 47 countries (70%) these were conducted between 2011 and 2014.

*World Bank income group indicates income group at the most recent survey.

DHS, Demographic Health Surveys; MICS, Multiple Indicator Cluster Surveys.

### Cross-sectional analyses of children without birth registration

Table 1 shows the percentage of children without birth registration based on caregiver report at the first and most recent survey. At the first survey, the smallest estimates of non-registration were similar across regions, and in five out of six regions were below 5%, which we defined as complete birth registration coverage. However, in South Asia, the smallest estimate was 58.9% (95% CI 57.8 to 59.9) in India in 2005. In contrast, the largest estimates of non-registration at the first survey varied widely by region: in Europe and Central Asia, the largest estimate of non-registration was 25.4% (95% CI 22.0 to 29.2) in Tajikistan in 2000; in South Asia it was 90.2% (95% CI 89.4 to 90.9) in Bangladesh in 2006 and in Sub-Saharan African non-registration was highest in Liberia in 2007 where 96.4% (95% CI 95.4 to 97.2) of children under five were unregistered. Estimates of non-registration from the most recent surveys also varied by region and were overall lower than estimates from the first survey. However, at the most recent survey, the percentage of children without birth registration was greater than 50% in 16 out of 67 countries. Online supplementary table S2 shows estimates and CIs for each survey by country.

**Figure 1:**
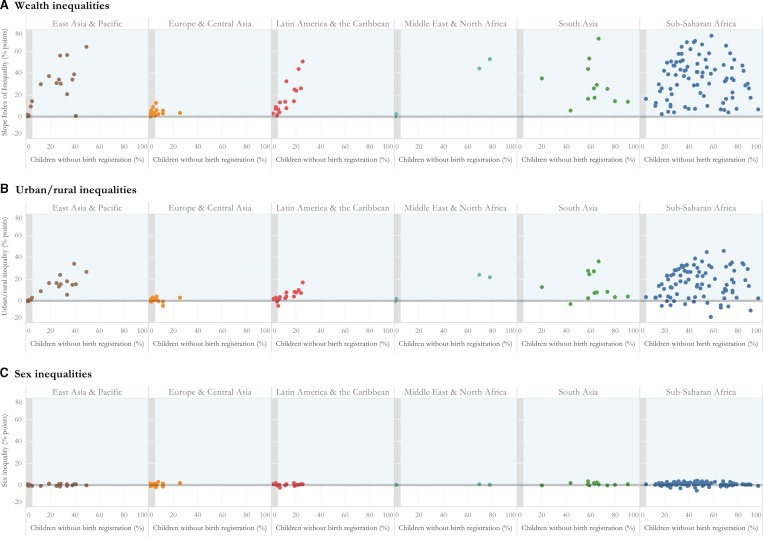
Wealth, urban/rural and sex inequalities in the % of children without birth registration in 173 national surveys stratified by region. Each dot is a survey (n=173) in a country. Zero is the null value and positive values in the shaded area represent inequalities. Wealth inequalities were estimated using the Slope Index of Inequality and five wealth quintiles. Urban/rural and sex inequalities were estimated by calculating the absolute difference in the % of children without birth registration residing in rural areas compared to urban areas, and among girls compared to boys.

Figure 1 shows wealth, urban rural and gender inequalities in birth registration for each survey. Large wealth and urban/rural inequalities were observed in surveys in every region except Europe and Central Asia and were largest in South Asia and Sub-Saharan Africa. The SII was significantly different from the null value of 0 representing no inequality in 129 out of 173 surveys which indicated that fewer children living in the poorest households compared with the wealthiest had their births registered. Among these surveys, wealth gaps were as large as 20 percentage points in in 89 surveys and larger than 50 percentage points in 26 surveys. The SII was greater than 70 percentage points in two countries: Nigeria in 2011 and Pakistan in 2012.

Urban/rural inequalities were not insubstantial: 105 out of 173 surveys had significant urban/rural inequalities. Non-registration was 20 percentage points or higher in rural areas compared with urban areas in 45 surveys. Niger in 2006 and 2010 was the only country with an absolute difference larger than 40 percentage points between urban and rural areas. In contrast, the percentage of children without birth registration was statistically significantly higher in urban areas compared with rural areas in six surveys. Online supplementary tables S3 and S4 show estimates and CIs wealth and urban/rural inequalities for each survey by country.

Gender inequalities in registration were observed in fewer surveys and were smaller in magnitude. In 17 surveys, the absolute difference in non-registration was statistically significantly higher among girls compared with boys, however the largest inequality was 3.9 percentage points in Mali in 2006. In five surveys, the reverse was observed, and non-registration was significantly higher among boys—the largest gap was 5.3 percentage points in Swaziland in 2014.

### Changes in the percentage of children without birth registration (1999-2016)

**Figure 2:**
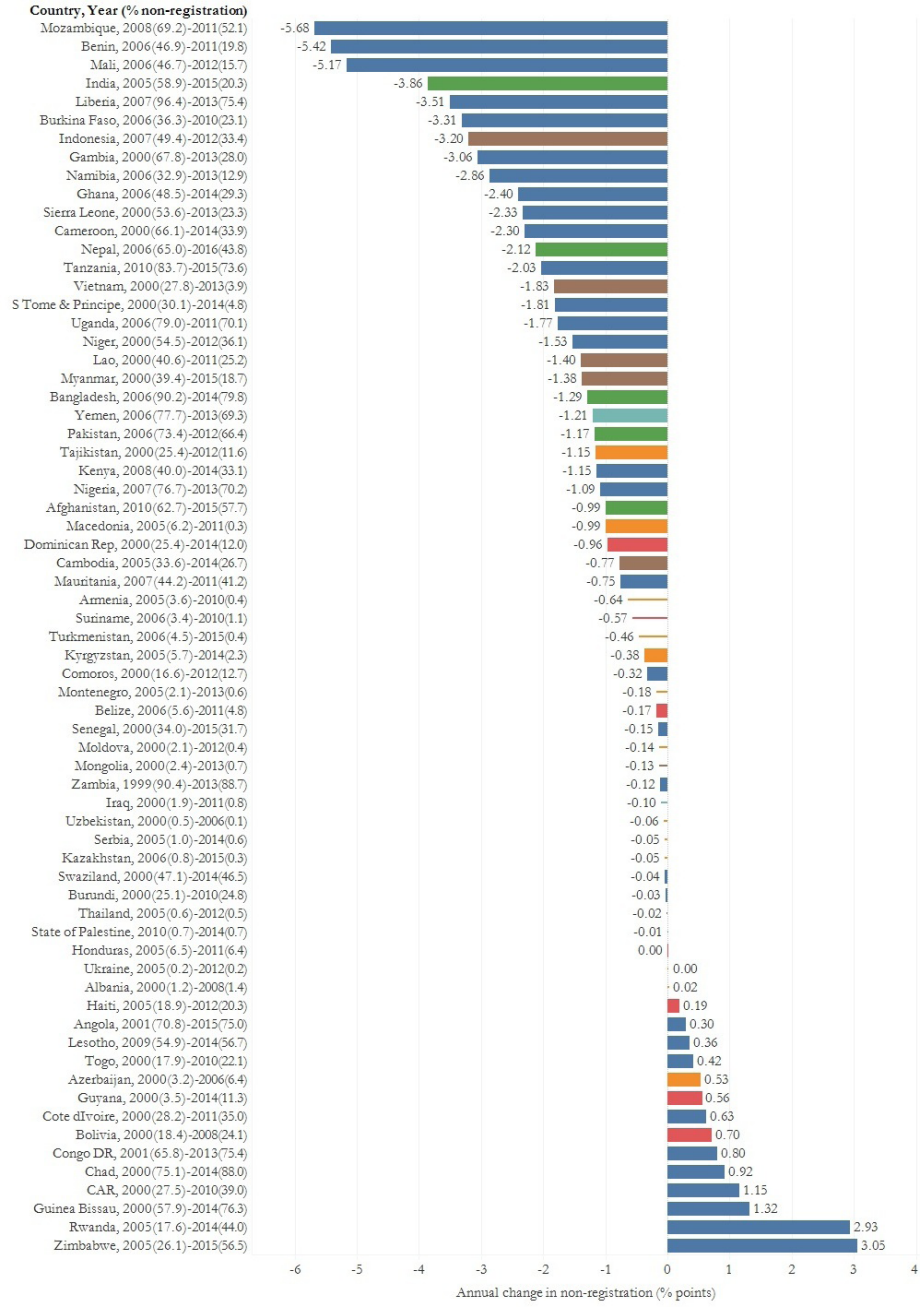
Annual change in the percentage of children without birth registration between the first and most recent survey in low-income and middle-income countries. Country information includes the year of the first and most recent survey used to calculate annual change and the % of children without birth registration at each time point. Lines represent the 14 countries which have achieved complete birth registration coverage (non-registration<=5%) and bars represent the 53 countries which have not achieved complete birth registration coverage.

Figure 2 shows changes in the percentage of children without birth registration for each country. A total of 14 countries, of which 9 were in Europe and Central Asia, had achieved complete birth registration coverage by the first survey and sustained this until the most recent survey. Among the 53 countries that had not achieved complete coverage, non-registration decreased in 39 countries and increased in 14 countries. Among the 32 countries in Sub-Saharan Africa included in the sample, non-registration increased in 10 and decreased in 22 countries.

Annual decreases in non-registration ranged from 5.7 percentage points in Mozambique to 0.001 percentage points in Honduras. A total of 26 countries achieved an annual decrease greater than 1 percentage point, and 8 of these countries achieved decreases greater than 3 percentage points. Annual increases in non-registration were smaller compared with decreases and ranged from 0.2 percentage point in Haiti to 3 percentage points in Zimbabwe. Annual increases in non-registration greater than 1 percentage point were observed in four countries. Online supplementary table S2 shows the magnitude and direction of total change in non-registration, annual change and changes between survey wave in all 67 countries.

**Figure 3:**
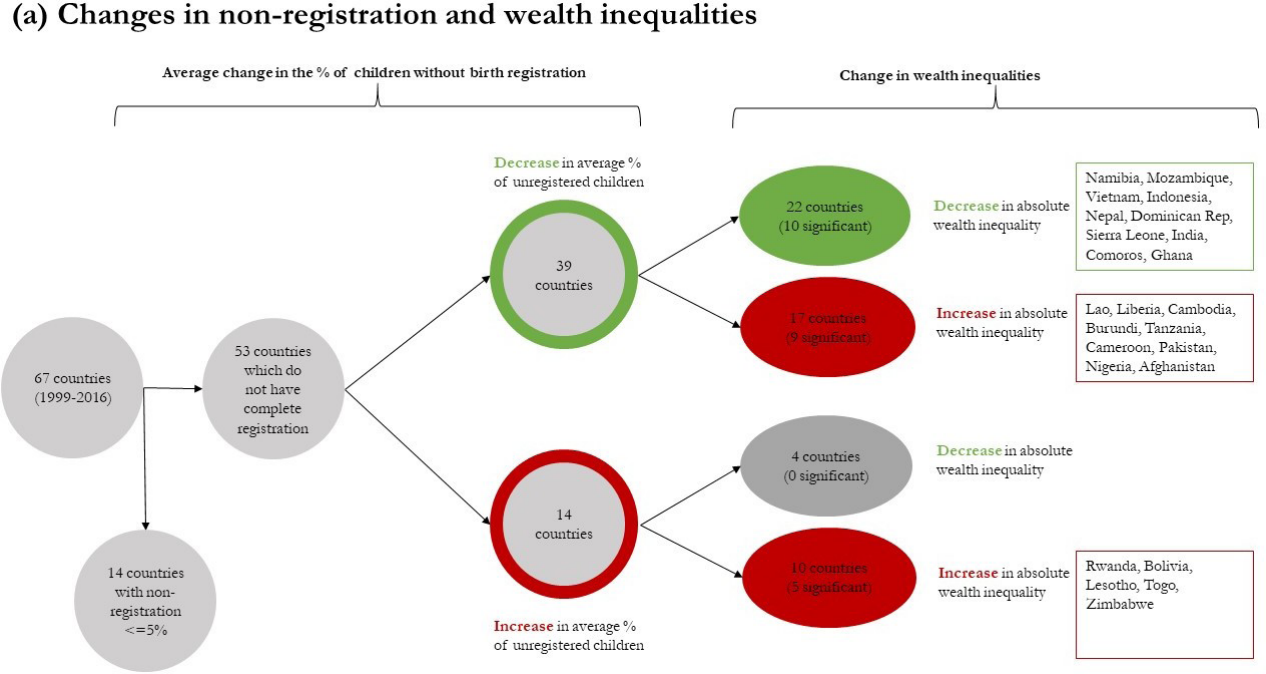
Change in (A) wealth and (B) urban/rural inequalities among countries which increased and decreased non-registration. Figure shows the number of countries which achieved changes in (a) wealth inequalities and (b) urban/rural inequalities relative to changes in non-registration.Notes: Supplementary Tables S3 and S4 show the magnitude and direction of total change in (a) SII and (b) urban/rural inequalities, annual change, and changes between survey wave in all 67 countries.

The flowcharts in figure 3 group countries according to whether reductions in both non-registration and inequalities were achieved or not and summarises concurrent changes in wealth (figure 3A) and urban/rural inequalities (figure 3B). Among the 53 countries, which did not have complete birth registration, 39 countries successfully decreased the percentage of children without birth registration. However, in most of these countries, statistically significant reductions in wealth inequalities were not observed: 10 out of 39 countries achieved significant reductions in wealth inequalities, and in 9 countries significant increases in wealth inequalities were observed. Similarly, in most of the 39 countries where non-registration decreased, urban/rural inequalities did not decline: 9 countries achieved significant reductions in urban/rural inequalities in 4 countries significant increases in urban/rural inequalities were observed.

Figure 3 also shows that among the 14 countries where the percentage of children without birth registration increased between the first and most recent survey, there were concurrent and significant increases in wealth inequalities in 5 countries and in urban/rural inequalities in 4 countries. None of the 14 countries where non-registration increased were able to achieve significant reductions in wealth and urban/rural inequalities. Online supplementary tables S2 and S3 show the magnitude and direction of total change in the SII and urban/rural inequalities, annual change and changes between survey wave in all 67 countries.

**Figure 4:**
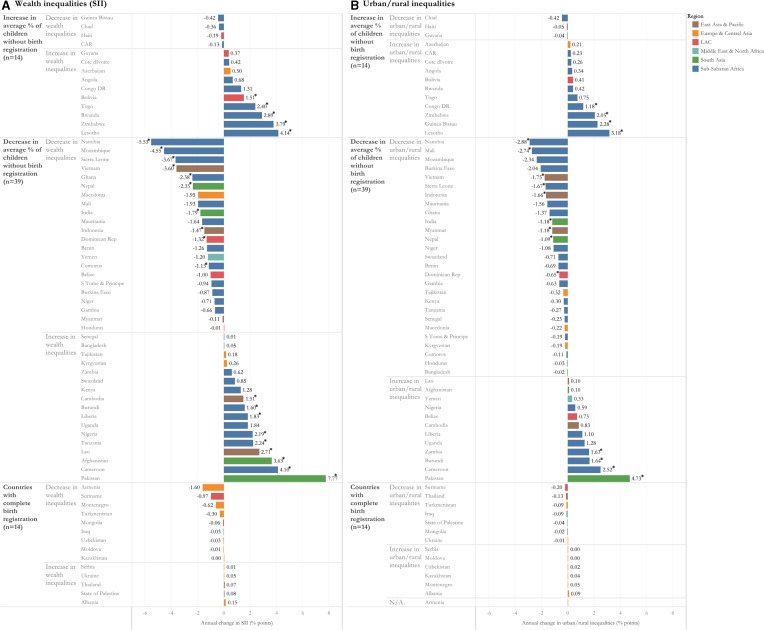
Magnitude of annual change in (A) wealth inequalities and (B) urban/rural inequalities among countries which increased and decreased non-registration.

Figure 4 shows the magnitude of annual change in inequalities for each of the groups in figure 3. Among the countries where non-registration increased, the largest increase in wealth inequalities was in Lesotho. Between 2006 and 2014, non-registration increased by 1.8 percentage points (0.36 percentage points per year) and wealth inequalities increased by 20.7 percentage points (4.1 percentage points per year), indicating that children in the poorest households faced the largest increase in non-registration. Among countries where non-registration decreased, the largest annual decline in wealth inequalities (5.5 percentage points) and urban/rural inequalities (2.9 percentage points) was in Namibia, while in Pakistan the on-average decline in non-registration was paired with the largest annual increase in wealth inequalities (7.8 percentage points) and urban/rural inequalities (4.7 percentage points). Countries with complete birth registration reduced any remaining wealth and urban/rural inequalities. Notably, there was significant correlation between annual change in wealth and urban/rural inequalities (correlation coefficient=0.82).

## Discussion

To our knowledge, this is the largest global study on birth registration, which combines data from 9.3 million children in 173 nationally representative DHS and MICS surveys across 67 LMICs countries to examine changes in averages and inequalities in birth registration during the Millenium Development Goal (MDG) and SDG period in a comparative and systematic way. We contend that the wealth and urban/rural inequalities in registration our findings reveal are unfair, unjust and avoidable and therefore represent inequities.^12–14^ Findings show that although 39 out of 67 countries were able to reduce the average percentage of children without birth registration between 1999 and 2016, such reductions did not occur alongside similar reductions in wealth or urban/rural inequities. Additionally, we show that gender inequities in birth registration at the national level were consistently small at each time point.

There are a number of limitations to this study. Although we rely on the most recent data available, the most recent surveys in our sample are from 2015 and may not reflect the most current coverage of birth registration. Notably, we do not examine access to birth certificates—documentary proof of registration—which, prior research indicates is likely to be lower than access to registration in many countries.^10 44^ The sample of countries included reflects (1) the focus of the DHS and MICS programme on LMICs, (2) when questions on birth registration were included and removed from these surveys especially since birth registration questions from national surveys are often removed as CRVS systems improve, and (3) which countries had more than one survey available. In response to these limitations, we refrained from pooling data to construct regional or global estimates, and we estimated annual change to make countries comparable. Given that the annual change approach treats all annual changes as the same regardless of when they occurred, we also estimate change between survey waves to partially address this limitation.

The DHS and MICS sampling approach and data structure could also affect our results. First, there could be some measurement error as caregivers are asked whether a child’s birth is registered with the ‘civil authorities’, a term which could differ in its interpretation across countries and across survey years, especially if the physical birth certificate is not available. However, this should not affect estimates within countries. Second, the MICS and DHS data used do not include children living outside households who are more likely to be marginalised and vulnerable and also do not include information on infant deaths, especially neonatal deaths, for whom birth registration is often overlooked.^6 44^ It is therefore likely that we underestimate the percentage of children living without birth registration and the invisibilities which drive this under-report will persist as long as household-based national surveys are the primary—and only—data source in LMICs to investigate birth registration. Third, wealth quintiles represent relative rather than absolute wealth, so the poorest quintile in one country could be wealthier than the second or third quintile in another country. Other data limitations that affected our analytic approach include the lack of data on child’s age at registration, making it challenging to estimate which children were registered in a given year and to estimate yearly trends in registration. There is also no information on birth registration among children above 5 years of age, on location of registration and on facility birth for children who are not living with their biological mothers: this limits the development of detailed subnational recommendations on where birth registration access could be improved. We also recognise that a registered birth and a functioning CRVS system may not translate to the publication and use of vital statistics. However, complete registration does represent an important step.

Finally, there are limitations in the choice of measure to estimate inequalities: absolute differences are sensitive to changes in the number of individuals in each stratification category and to effect modification by time.^43 45^ However, in most countries, the male/female ratio is close to even and survey sampling should help account for urban/rural migration. Finally, all estimates are at the national level, which may obscure large within country differences in birth registration by region or inequities within-regions, particularly by gender and particularly in countries with large sex ratios at birth. Future work could examine subnational changes in birth registration and inequities at the regional or state level to guide the targeting of efforts within countries to improve registration.

However, our findings make two key contributions to prior research that documents global improvements in access to birth registration.^2 3^ First, we show that improvements in access to birth registration were neither ubiquitous nor universal, as evidenced by the 14 countries where non-registration increased and by the 16 countries where, even at the most recent survey, 1 in 2 children under five did not have their births registered. Second, our findings suggest that even improvements in ‘on average’ access to birth registration should be interpreted with caution and through the lens of equity-oriented analyses which reveal that across the 39 countries where non-registration declined, improvements in many countries were unfairly and unevenly distributed.

Our findings echo prior work on how national averages can mask health inequities,^46^ and how large inequities in child health outcomes and access can persist even in the context of increasing national coverage.^43 47^ Prior research has also demonstrated how absolute health inequities increase in the short term and how by the time coverage improves among the most deprived groups, coverage among the most privileged groups is already close to 100%.^48^ Similarly, historical research in high-income countries suggests that the expansion of birth registration was unequal and documents how minority and marginalised populations were initially excluded.^49–51^ Our results raise questions about whether the path to universal coverage of birth registration is through large wealth and urban/rural inequities: the majority of surveys in the sample underscore that children in poor households and rural areas are more likely to be living without the benefits of birth registration, and that improvements are slower among children in poor and rural households. Critically, our results also show that these inequities can be reduced if not eliminated and are therefore not inevitable.

In addition to the 14 countries in our sample that achieved complete birth registration and also eliminated wealth, urban/rural other inequities, 10 countries (Namibia, Mozambique, Vietnam, Indonesia, Nepal, Dominican Republic, Sierra Leone, India, Comoros, Ghana) were able to achieve a concurrent decline in non-registration and in wealth inequities. Of note, there were 6 countries (Namibia, Vietnam, Sierra Leone, Indonesia, India, Dominican Rep) where both wealth and urban/rural inequities declined. However, it is important to note that even among this sample, improvements varied. For example, wealth inequities in Indonesia decreased on average by 1.5 percentage points per year from a 64.0 (2007) to a 56.7 (2012) percentage point difference between children in the poorest and wealthiest households. Although this decline is statistically significant, children from the poorest two wealth quintiles were still almost half as likely to be registered in 2012. In contrast, Sierra Leone achieved a 3.7 percentage point per year decline and wealth inequities fell from 52.5 (2005) to 4.7 (2013) percentage points. Both declines were significant but varied greatly in magnitude. Lessons from these countries could be instructive in understanding approaches to concurrently improve registration and reduce inequities. Further work is needed to untangle the policies and initiatives underpinning these changes, including whether this is the unintended effects of the status quo or a result of policies that were aiming to improve averages or specifically reduce inequities. A brief review of the literature indicates that the following factors can improve birth registration: reducing registration fees; linking registration to the health and education systems; expanding welfare and social protection; strengthening the design of the CRVS system (e.g., through process mapping and digitisation); harmonising parallel identification systems and removing policies and laws which (1) require the marriage certificates of parents to register a child, (2) require a father to be present and (3) discriminate against ethnic minorities especially in the context of providing nationality.^11 52–58^

Our findings make visible, and quantify, the large wealth and urban/rural inequities which most countries, even those that have improved birth registration, must tackle. These findings contribute to: critical scholarship on who is excluded from definitions and counts of a population^59^; the growing literature on the importance of studying changes in health inequities over time^60–63^ and to research arguing for the importance of public health data systems which allow health equity to be monitored to guide the development of equity-oriented policies, programme and practices.^46 64–66^ More specifically, our findings support current research and policy calling attention to the need to improve CRVS systems which remain underfunded, underprioritised and underdeveloped in many countries,^3–5^ have made modest progress,^5 21^ and continue to exclude marginalised populations.^11^ The recent galvanisation of resources and commitments made by international agencies, governments and local organisations have led to a multiplicity of efforts to improve the quality, availability and use of vital statistics for public health^21^ and assess whether ‘every child is counted’ in the context of the SDGs.^39^ It is in this context that we bring attention to the need for governments and development partners to assess inequities in civil registration and to ensure that equity-oriented approaches are central to efforts to improve birth registration.

To this end, we also show that the analysis of the birth registration indicator in national surveys provides important insights into exclusions in civil registries: health statistics from civil registries in countries with large inequities in birth registration are likely to systematically mis-estimate infant mortality rates as children from poor and rural households are less likely to be counted. However, such analyses of survey data are often based on data which are several years old and do not obviate the need for direct assessments of CRVS coverage, quality and functioning, which are current and can guide improvements. For example, the vital statistics performance index indicator, which is solely based on death registration, has been used to measure the performance of CRVS systems,^5 67^ and has more recently been adapted to assess birth registration data quality and whether data on birth weight, live birth order, maternal age and sex of child are captured.^44^ Such efforts continue to be important especially since the ultimate goal is to develop inclusive national data systems which make redundant the use of national surveys to measure birth registration and which ensure children and adults have equitable access to birth registration.

## Conclusion

Findings reveal that the wealth and urban/rural inequities in birth registration have persisted in most countries and highlight the importance of monitoring changes in equity to improve birth registration systems and to achieve SDG 16.9. Efforts to improve birth registration should strive to improve averages and actively reduce the unfair and unequal distribution of birth registration. Equity analyses can guide national policy, technical assistance to countries and provide insights into how inclusive CRVS systems are.

## References

[1] United Nations Sustainable development goals 2016, 2017 Available: https://sustainabledevelopment.un.org/sdgs [Accessed 08 Feb 2017].

[2] UNICEF Every child's birth right: inequities and trends in birth registration. New York: UNICEF, 2013.

[3] World Bank, WHO Global civil registration and vital statistics. scaling up investment plan 2015-2024. Washington DC and Geneva: World Bank and WHO, 2014.

[4] Cappa C, Gregson K, Wardlaw T, et al Birth registration: a child's passport to protection. Lancet Glob Health 2014;2:e67–8. 10.1016/S2214-109X(13)70180-3 25104657

[5] Mikkelsen L, Phillips DE, AbouZahr C, et al A global assessment of civil registration and vital statistics systems: monitoring data quality and progress. The Lancet 2015;386:1395–406. 10.1016/S0140-6736(15)60171-4 25971218

[6] AbouZahr C, de Savigny D, Mikkelsen L, et al Civil registration and vital statistics: progress in the data revolution for counting and accountability. The Lancet 2015;386:1373–85. 10.1016/S0140-6736(15)60173-8 PMC7753937 25971224

[7] UNICEF The state of the World’s children 2017: children in a digital world. New York: UNICEF, 2017.

[8] Adi AE, Abdu T, Khan A, et al Understanding whose births get registered: a cross sectional study in Bauchi and cross river states, Nigeria. BMC Res Notes 2015;8:79 10.1186/s13104-015-1026-y 25879591 PMC4369829

[9] Amo-Adjei J, Annim SK Socioeconomic determinants of birth registration in Ghana. BMC Int Health Hum Rights 2015;15:14 10.1186/s12914-015-0053-z 26072313 PMC4465725

[10] Bhatia A, Ferreira LZ, Barros AJD, et al Who and where are the uncounted children? inequalities in birth certificate coverage among children under five years in 94 countries using nationally representative household surveys. Int J Equity Health 2017;16:148 10.1186/s12939-017-0635-6 28821291 PMC5562988

[11] Hunter W, Documents I Identity documents, welfare enhancement, and group Empowerment in the global South. J Dev Stud 2019;55:366–83. 10.1080/00220388.2018.1451637

[12] Braveman P Health disparities and health equity: concepts and measurement. Annu Rev Public Health 2006;27:167–94. 10.1146/annurev.publhealth.27.021405.102103 16533114

[13] Whitehead M The concepts and principles of equity and health. Int J Health Serv 1992;22:429–45. 10.2190/986L-LHQ6-2VTE-YRRN 1644507

[14] Commission on Social Determinants of Health Closing the gap in a generation: health equity through action on the social determinants of health. final report of the Commission on social determinants of health. Geneva: World Health Organization, 2008.

[15] Department of Economic and Social Affairs Principles and recommendations for a vital statistics system. statistical papers. New York United Nations, 2014.

[16] Breckenridge KD, Szreter S Registration and recognition: documenting the person in world history. Oxford University Press: Oxford, 2012.

[17] Szreter S The right of registration: development, identity registration, and social Security—A historical perspective. World Dev 2007;35:67–86. 10.1016/j.worlddev.2006.09.004

[18] Brolan CE, Gouda HN, AbouZahr C, et al Beyond health: five global policy metaphors for civil registration and vital statistics. The Lancet 2017;389:1084–5. 10.1016/S0140-6736(17)30753-5 28322806

[19] Abouzahr C, Stein C, Chapman N, et al A development imperative: civil registration and vital statistics systems in the Asia-Pacific region. Asia Pac Popul J 2012;29:9–37. 10.18356/b528561c-en

[20] Setel PW, Macfarlane SB, Szreter S, et al A scandal of invisibility: making everyone count by counting everyone. The Lancet 2007;370:1569–77. 10.1016/S0140-6736(07)61307-5 17992727

[21] Mahapatra P, Shibuya K, Lopez AD, et al Civil registration systems and vital statistics: successes and missed opportunities. The Lancet 2007;370:1653–63. 10.1016/S0140-6736(07)61308-7 18029006

[22] Bhutta ZA, Black RE, maternal G Newborn, and child health-so near and yet so far. N Engl J Med 2013;369:2226–35. 24304052 10.1056/NEJMra1111853

[23] Victora CG Causes of child deaths: looking to the future. The Lancet 2015;385:398–9. 10.1016/S0140-6736(14)61695-0 25282518

[24] UN General Assemby Convention on the rights of the child. New York: United Nations General Assembly, 2014.

[25] Bhabha J From citizen to migrant: the scope of child Statelessness in the twenty-first century. children without a state: a global human rights challenge. Cambridge: MIT university press 2011:1–39.

[26] Plan International Birth registration in emergencies: a review of best practices in humanitarian action Surrey: plan international 2014.

[27] Institute on Statelessness and Inclusion Statelessness, human rights and the sustainable development agenda. Eindhoven, The Netherlands: Institute on Statelessness and Inclusion, 2017.

[28] Jayaraman J, Roberts GJ, Wong HM, et al Ages of legal importance: implications in relation to birth registration and age assessment practices. Med Sci Law 2016;56:77–82. 10.1177/0025802415590172 26101440

[29] Comandini O, Cabras S, Marini E Birth registration and child undernutrition in sub-Saharan Africa. Public Health Nutr 2016;19:1757–67. 10.1017/S136898001500333X 26669828 PMC10271021

[30] Covering every birth and death: Improving civil registration and vital statistics (CRVS) Report of the technical discussions 16–17 June 2014. Sixty-sixth session of the regional Committee for who south-east Asia. New Delhi: World Health Organization Regional Office for South-East Asia, 2014.

[31] Hanmer L, Elefante M The role of identification in ending child marriage. Washington DC: The World Bank, 2016.

[32] Apland K, Hamilton C, Blitz BK, et al Birth registration and children's rights. Plan International: Woking, Surrey, 2014.

[33] Dunning C, Gelb A, Raghavan S, et al Legal identity, and the post-2015 agenda. Washington, DC: Center for Global Development, 2014.

[34] Jeong J, Bhatia A, Fink G Associations between birth registration and early child growth and development: evidence from 31 low- and middle-income countries. BMC Public Health 2018;18:673 10.1186/s12889-018-5598-z 29848302 PMC5977554

[35] Phillips DE, AbouZahr C, Lopez AD, et al Are well functioning civil registration and vital statistics systems associated with better health outcomes? The Lancet 2015;386:1386–94. 10.1016/S0140-6736(15)60172-6 25971222

[36] Brito S, Corbacho A, Osorio R Does birth under-registration reduce childhood immunization? Evidence from the Dominican Republic. Health Econ Rev 2017;7:14 10.1186/s13561-017-0149-3 28337738 PMC5364131

[37] Hancioglu A, Arnold F Measuring coverage in MNCH: tracking progress in health for women and children using DHS and MICs household surveys. PLoS Med 2013;10:e1001391 10.1371/journal.pmed.1001391 23667333 PMC3646216

[38] Victora CG, Requejo JH, Barros AJD, et al Countdown to 2015: a decade of tracking progress for maternal, newborn, and child survival. The Lancet 2016;387:2049–59. 10.1016/S0140-6736(15)00519-X PMC7613171 26477328

[39] UNICEF Is every child counted? status of data for children in the SDGs. New York: UNICEF, 2017.

[40] World Bank World bank country and lending groups: world bank. Available: https://datahelpdesk.worldbank.org/knowledgebase/articles/906519-world-bank-country-and-lending-groups [Accessed 10 Feb 2019].

[41] Rutstein SO, Johnson K The DHS wealth index. DHS comparative reports. Calverton, Maryland, USA, 2004.

[42] Rutstein SO The DHS wealth index: approaches for rural and urban areas. DHS working papers. Calverton, MD: Macro International Inc, 2008.

[43] Barros AJD, Victora CG Measuring coverage in MNCH: determining and interpreting inequalities in coverage of maternal, newborn, and child health interventions. PLoS Med 2013;10:e1001390 10.1371/journal.pmed.1001390 23667332 PMC3646214

[44] Phillips DE, Adair T, Lopez AD How useful are registered birth statistics for health and social policy? a global systematic assessment of the availability and quality of birth registration data. Popul Health Metr 2018;16:21 10.1186/s12963-018-0180-6 30587201 PMC6307230

[45] Chen JT, Beckfield J, Waterman PD, et al Can changes in the distributions of and associations between education and income bias temporal comparisons of health disparities? an exploration with causal graphs and simulations. Am J Epidemiol 2013;177:870–81. 10.1093/aje/kwt041 23568593 PMC4023297

[46] Hosseinpoor AR, Bergen N Health Inequality Monitoring: A Practical Application of Population Health Monitoring : Verschuuren M, van Oers H, Population health monitoring: climbing the information pyramid. Cham: Springer International Publishing, 2019: 151–73.

[47] WHO Handbook on health inequality monitoring: with a special focus on low-and middle-income countries. Geneva: World Health Organization, 2013.

[48] Victora CG, Joseph G, Silva ICM, et al The inverse equity hypothesis: analyses of institutional deliveries in 286 national surveys. Am J Public Health 2018;108:464–71. 10.2105/AJPH.2017.304277 29470118 PMC5844402

[49] Krieger N, Fee E Measuring social inequalities in health in the United States: a historical review, 1900-1950. Int J Health Serv 1996;26:391–418. 10.2190/B3AH-Q5KE-VBGF-NC74 8840195

[50] Shapiro S Development of birth registration and birth statistics in the United States. Popul Stud 1950;4:86–111. 10.1080/00324728.1950.10415506

[51] Hetzel AM History and organization of the vital statistics system 1950;1:1–19.

[52] Muzzi M Good practices in integrating birth registration into health systems (2000–2009); case studies. Bangladesh, Brazil, the Gambia and Delhi, India New York: UNICEF, 2010.

[53] Plan International Innovations in birth registration. Woking: Plan International, 2017.

[54] World Bank Incentives for improving birth registration coverage: a review of the literature. Washington, DC: World Bank License: Creative Commons Attribution 3.0 IGO (CC BY 3.0 IGO), 2016.

[55] Hunter W, Brill R “Documents, Please”: Advances in Social Protection and Birth Certification in the Developing World. World Politics 2016;68:191–228.

[56] CRVS Knowledge Gateway Developing an enterprise architecture and business process mapping for CRVS systems. Melbourne: University of Melbourne, 2018 https://crvsgateway.info/Developing-an-enterprise-architecture-and-business-process-mapping-for-CRVS-systems~432

[57] Plan International and Jembi Health Systems Civil registration and vital statistics Digitisation Guidebook African development bank for the African programme for the accelerated improvement of civil registration and vital statistics 2017.

[58] Suthar AB, Khalifa A, Yin S, et al Evaluation of approaches to strengthen civil registration and vital statistics systems: a systematic review and synthesis of policies in 25 countries. PLoS Med 2019;16:e1002929 10.1371/journal.pmed.1002929 31560684 PMC6764661

[59] Krieger N Who and what is a "population"? Historical debates, current controversies, and implications for understanding "population health" and rectifying health inequities. Milbank Q 2012;90:634–81. 10.1111/j.1468-0009.2012.00678.x 23216426 PMC3530737

[60] Restrepo-Méndez MC, Barros AJD, Black RE, et al Time trends in socio-economic inequalities in stunting prevalence: analyses of repeated national surveys. Public Health Nutr 2015;18:2097–104. 10.1017/S1368980014002924 25521530 PMC4909139

[61] Restrepo-Méndez MC, Barros AJD, Requejo J, et al Progress in reducing inequalities in reproductive, maternal, newborn,' and child health in Latin America and the Caribbean: an unfinished agenda. Rev Panam Salud Publica 2015;38:9–16. 26506316

[62] Restrepo-Méndez MC, Barros AJ, Wong KL, et al Inequalities in full immunization coverage: trends in low- and middle-income countries. Bull World Health Organ 2016;94:794–805. 10.2471/BLT.15.162172 27821882 PMC5096343

[63] Alkenbrack S, Chaitkin M, Zeng W, et al Did equity of reproductive and maternal health service coverage increase during the mdg era? an analysis of trends and determinants across 74 low- and middle-income countries. PLoS One 2015;10:e0134905 10.1371/journal.pone.0134905 26331846 PMC4558013

[64] Hosseinpoor AR, Bergen N, Schlotheuber A Promoting health equity: who health inequality monitoring at global and national levels. Glob Health Action 2015;8:29034 10.3402/gha.v8.29034 26387506 PMC4576419

[65] CSDH WHO Closing the gap in a generation: health equity through action on the social determinants of health. final report of the Commission on social determinants of health. Geneva: World Health Organization, 2008. 10.1016/S0140-6736(08)61690-6 18994664

[66] Hosseinpoor AR, Bergen N, Schlotheuber A, et al National health inequality monitoring: current challenges and opportunities. Glob Health Action 2018;11:70–4. 10.1080/16549716.2017.1392216 PMC5827767 29460696

[67] Phillips DE, Lozano R, Naghavi M, et al A composite metric for assessing data on mortality and causes of death: the vital statistics performance index. Popul Health Metr 2014;12:14. 24982595 10.1186/1478-7954-12-14 PMC4060759

