# Supplementary material for: Are inequities decreasing? Birth registration for children under five in low-income and middle-income countries, 1999–2016
Source: BMJ Glob Health. 2019 Dec 16;4(6):e001926. doi: 10.1136/bmjgh-2019-001926 (PMC6936526; doi:10.1136/bmjgh-2019-001926)
Supplement: Supplementary data [file bmjgh-2019-001926supp001.pdf]

Supplementary Material

Are inequities decreasing? Birth registration for children under five in low- and middle-income countries, 1999 to 2016

Figure

Figure S1: Countries and surveys included in the sample stratified by region and (a) World Bank income group and (b) survey wave

Tables

Table S1: Description of birth registration questions in MICS and DHS Surveys (1999-2016)

Table S2: Change in the percentage of children under five whose birth is not registered in 67 countries (1999-2016)

Table S3: Change in wealth inequalities in the percentage of children under five whose birth is not registered in 67 countries (1999-2016)

Table S4: Change in urban/rural inequalities in the percentage of children under five whose birth is not registered in 67 countries (1999-2016)

Figure S1: Countries and surveys included in the sample stratified by region and (a) World Bank income group and (b) survey wave

Region	(a) Countries			(b) Surveys Included	
	Total	Included	Countries stratified by World Bank Income Groups	Total	Surveys included (n) by wave
East Asia & Pacific	38	7	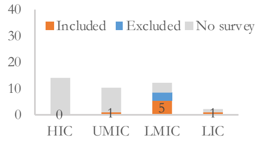	20	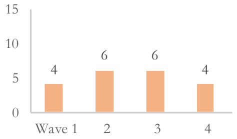
Europe & Central Asia	58	13	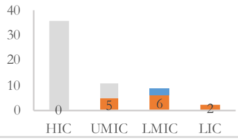	31	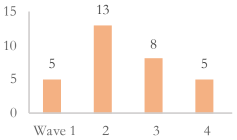
Latin America & Caribbean	42	7	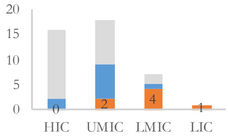	17	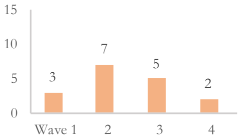
Middle East & North Africa	21	3	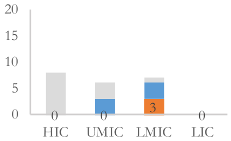	6	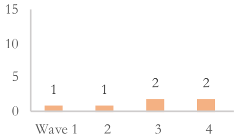
North America	3	0	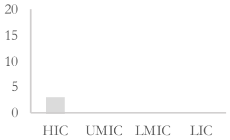	0	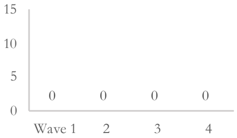
South Asia	8	5	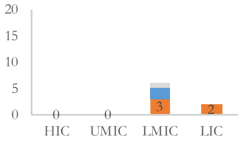	12	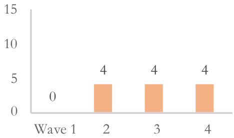
Sub-Saharan Africa	48	32	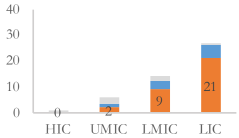	87	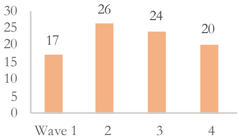
Total included	N/A	67	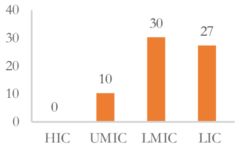	173	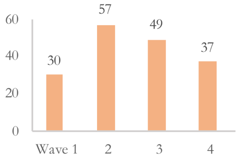

Table shows (a) the total number of countries with available data and the sample of countries stratified by region and income group which meet the inclusion criteria (two or more surveys with data on birth registration; covariate data on: sex of the child, household wealth quintiles, urban/rural residence; and, survey weights) and (b) surveys included by survey wave stratified by region. Survey waves defined as: Wave 1 (<2004); Wave 2 (2004-2007); Wave 3 (2009-2012); Wave 4 (2013+)
World Bank regional classifications used to group countries. World Bank Income Groups include: High Income (HIC), Low income (LIC), Lower-middle income (LMIC), Upper-middle income (UMIC). For countries without surveys, the 2017 World Bank Income Group was used. For countries with surveys, the World Bank Income Group is at the time of the most recent survey.

Table S1: Description of birth registration questions in MICS and DHS Surveys (1999-2016)

DHS

Household questionnaire Phase 5 (2003-2008)

IF AGE 0-4 YEARS<br><br>Has (NAME)'s birth ever been registered with the civil authority?<br><br>1 = HAS CERTIFICATE<br>2 = REGISTERED<br>3 = NEITHER<br>8 = DON'T KNOW

Household questionnaire Phase 6 (2008-2013)

IF AGE 0-4 YEARS<br><br>Does (NAME) have a birth certificate?<br><br>IF NO, PROBE:<br>Has (NAME)'s birth ever been registered with the civil authority?<br><br>1 = HAS CERTIFICATE<br>2 = REGISTERED<br>3 = NEITHER<br>8 = DON'T KNOW

Household questionnaire Phase 6 (2003-2018)

IF AGE 0-4 YEARS<br><br>Does (NAME) have a birth certificate?<br><br>IF NO, PROBE:<br>Has (NAME)'s birth ever been registered with the civil authority?<br><br>1 = HAS CERTIFICATE<br>2 = REGISTERED<br>3 = NEITHER<br>8 = DON'T KNOW

MICS 2

4. DOES (*name*) HAVE A BIRTH CERTIFICATE?<br>MAY I SEE IT?<br><br>*If certificate is presented, verify reported birth date.*<br>*If no birth certificate is presented, try to verify date using another document (health card, etc.).*<br>*Correct stated age, if necessary.*	Yes, seen .... 1<br>Yes, not seen .... 2<br>No .... 3<br><br>DK........ 9	1⇨Q.8
5. *If no birth certificate is shown, ask*:<br><br>HAS (*name's*) BIRTH BEEN REGISTERED?	Yes .... 1<br>No .... 2<br>DK........ 9	1⇨Q.8<br><br>9⇨Q.7

MICS 3

BR1. DOES (*name*) HAVE A BIRTH CERTIFICATE?<br>MAY I SEE IT?	Yes, seen ..... 1<br>Yes, not seen ..... 2<br>No ..... 3<br><br>DK..... 8	1⇨BR5
BR2. HAS (*name's*) BIRTH BEEN REGISTERED WITH THE CIVIL AUTHORITIES?	Yes ..... 1<br>No ..... 2<br>DK..... 8	1⇨BR5<br><br>8⇨BR4

MICS 4

BR1. DOES (*name*) HAVE A BIRTH CERTIFICATE?<br><br>*If yes, ask:*<br>MAY I SEE IT?	Yes, seen..... 1<br>Yes, not seen..... 2<br>No ..... 3<br>DK 8	1⇨NEXT MODULE<br>2⇨NEXT MODULE
BR2. HAS (*name*)'S BIRTH BEEN REGISTERED WITH THE CIVIL AUTHORITIES?	Yes..... 1<br>No ..... 2<br>DK 8	1⇨NEXT MODULE

MICS 5

**BR1**. DOES *(name)* HAVE A BIRTH CERTIFICATE?<br><br>*If yes, ask:*<br>MAY I SEE IT?	Yes, seen ..... 1<br>Yes, not seen..... 2<br>No ..... 3<br>DK 8	1⇨Next Module<br>2⇨Next Module
**BR2**. HAS (*name*)'S BIRTH BEEN REGISTERED WITH ***the civil authorities***?	Yes ..... 1<br><br>No ..... 2<br>DK 8	1⇨Next Module

Table S2: Change in the percentage of children under five whose birth is not registered in 67 countries (1999-2016)

Table S2: Change in the percentage of children under five whose birth is not registered in 67 countries (1999-2016)

Region	Country	Change in % of children under five whose birth is not registered between first and most recent survey							Percentage of children under five whose birth is not registered					Percentage of children under five whose birth is not registered				
		First survey		Most recent survey		Change			Wave 1 (1999-2004)					Wave 2 (2004-2008)				
		Year	Estimate	Year	Estimate	Years (n)	Total change	Annual change	Year	Estimate	95% CI		Children under 5 (n)	Year	Estimate	95% CI		Children under 5 (n)
East Asia & Pacific	Thailand	2005	0.62	2012	0.48	7	-0.14	-0.02						2005	0.62	0.40	0.97	4837680
	Mongolia	2000	2.38	2013	0.70	13	-1.68	-0.13	2000	2.38	1.96	2.88	6184	2005	1.67	1.26	2.20	3547
	Vietnam	2000	27.76	2013	3.94	13	-23.82	-1.83	2000	27.76	22.90	33.20	3105	2006	12.26	9.72	15.34	2677
	Cambodia	2005	33.61	2014	26.69	9	-6.92	-0.77						2005	33.61	31.59	35.69	7794
	Myanmar	2000	39.35	2015	18.71	15	-20.64	-1.38	2000	39.35	35.68	43.15	14443					
	Lao	2000	40.61	2011	25.24	11	-15.38	-1.40	2000	40.61	36.38	44.99	4610	2006	28.53	25.75	31.47	4136
	Indonesia	2007	49.36	2012	33.36	5	-16.00	-3.20						2007	49.36	47.55	51.17	16625
Europe & Central Asia	Ukraine	2005	0.19	2012	0.21	7	0.01	0.00						2005	0.19	0.06	0.62	3049
	Uzbekistan	2000	0.48	2006	0.09	6	-0.39	-0.06	2000	0.48	0.28	0.82	3349	2006	0.09	0.03	0.28	4986
	Kazakhstan	2006	0.77	2015	0.33	9	-0.45	-0.05						2006	0.77	0.50	1.18	4415
	Serbia	2005	1.04	2014	0.58	9	-0.46	-0.05						2005	1.04	0.70	1.55	3687
	Albania	2000	1.17	2008	1.35	8	0.18	0.02	2000	1.17	0.66	2.06	1454	2008	1.35	0.83	2.19	1596
	Montenegro	2005	2.05	2013	0.59	8	-1.46	-0.18						2005	2.05	0.76	5.39	1028
	Moldova	2000	2.13	2012	0.43	12	-1.71	-0.14	2000	2.13	1.45	3.13	1656	2005	7.66	6.19	9.45	1704
	Azerbaijan	2000	3.20	2006	6.38	6	3.18	0.53	2000	3.20	2.21	4.62	1875	2006	6.38	4.80	8.42	2210
	Armenia	2005	3.61	2010	0.41	5	-3.20	-0.64						2005	3.61	2.08	6.21	1526
	Turkmenistan	2006	4.52	2015	0.42	9	-4.10	-0.46						2006	4.52	3.35	6.08	2076
	Kyrgyzstan	2005	5.67	2014	2.27	9	-3.40	-0.38						2005	5.67	3.72	8.55	2987
	Macedonia	2005	6.19	2011	0.26	6	-5.93	-0.99						2005	6.19	3.97	9.53	4547
	Tajikistan	2000	25.43	2012	11.58	12	-13.85	-1.15	2000	25.43	22.04	29.16	3535	2005	11.74	9.94	13.81	4273
Latin America and the Caribbean	Suriname	2006	3.39	2010	1.10	4	-2.29	-0.57						2006	3.39	2.47	4.64	2257
	Guyana	2000	3.48	2014	11.26	14	7.77	0.56	2000	3.48	2.49	4.85	2672	2006	6.75	5.50	8.26	2499
	Belize	2006	5.62	2011	4.76	5	-0.86	-0.17						2006	5.62	3.92	8.01	797
	Honduras	2005	6.46	2011	6.45	6	-0.01	0.00						2005	6.46	5.90	7.06	11069
	Bolivia	2000	18.44	2008	24.08	8	5.64	0.70	2000	18.44	16.62	20.40	3156	2008	24.08	22.80	25.40	9118
	Haiti	2005	18.92	2012	20.25	7	1.33	0.19						2005	18.92	17.25	20.71	6001
	Dominican Rep	2000	25.41	2014	11.97	14	-13.44	-0.96	2000	25.41	22.03	29.10	1993	2007	22.22	20.72	23.79	11694
Middle East & North Africa	State of Palestine	2010	0.69	2014	0.66	4	-0.03	-0.01										
	Iraq	2000	1.88	2011	0.77	11	-1.11	-0.10	2000	1.88	1.60	2.21	14553					
	Yemen	2006	77.75	2013	69.30	7	-8.45	-1.21						2006	77.75	73.36	81.60	3781
South Asia	India	2005	58.85	2015	20.27	10	-38.58	-3.86						2005	58.85	57.78	59.91	55896
	Afghanistan	2010	62.66	2015	57.70	5	-4.96	-0.99										
	Nepal	2006	65.03	2016	43.79	10	-21.24	-2.12						2006	65.03	61.68	68.25	5440
	Pakistan	2006	73.42	2012	66.39	6	-7.03	-1.17						2006	73.42	71.16	75.56	8760
	Bangladesh	2006	90.15	2014	79.84	8	-10.31	-1.29						2006	90.15	89.41	90.85	31562

Table S2: Change in the percentage of children under five whose birth is not registered in 67 countries (1999-2016)

Region	Country	Percentage of children under five whose birth is not registered Wave 3 (2009-2012)					Percentage of children under five whose birth is not registered Wave 4 (2013-2015)					Interval change in percentage of children under five whose birth is not registered (% points)					
		Year	Estimate	95% CI		Children under 5 (n)	Year	Estimate	95% CI		Children under 5 (n)	Wave 1 to 2	Wave 2 to 3	Wave 3 to 4	Wave 1 to 4	Wave 2 to 4	Wave 1 to 3
East Asia & Pacific	Thailand	2012	0.48	0.31	0.76	9716							-0.14				
	Mongolia	2010	1.03	0.72	1.48	3956	2013	0.70	0.52	0.95	6054	-0.70905	-0.64	-0.33	-1.68	-0.97	-1.35
	Vietnam	2010	5.03	3.88	6.50	3678	2013	3.94	3.16	4.89	3316	-15.5007	-7.23	-1.09	-23.82	-8.32	-22.73
	Cambodia	2010	37.94	35.97	39.95	8123	2014	26.69	24.67	28.81	7806		4.34	-11.26		-6.92	
	Myanmar						2015	18.71	16.22	21.49	4634				-20.64		
	Lao	2011	25.24	23.62	26.92	11067						-12.0869	-3.29				-15.38
	Indonesia	2012	33.36	31.87	34.88	16785							-16.00				
Europe & Central Asia	Ukraine	2012	0.21	0.05	0.85	4379							0.01				
	Uzbekistan											-0.38549					
	Kazakhstan	2010	0.26	0.15	0.43	5181	2015	0.33	0.19	0.57	5172		-0.52	0.07		-0.45	
	Serbia	2010	1.08	0.45	2.55	3374	2014	0.58	0.28	1.19	2720		0.04	-0.50		-0.46	
	Albania											0.18					
	Montenegro						2013	0.59	0.26	1.36	1420					-1.46	
	Moldova	2012	0.43	0.21	0.89	1869						5.53	-7.23				-1.71
	Azerbaijan											3.18					
	Armenia	2010	0.41	0.17	1.04	1480							-3.20				
	Turkmenistan						2015	0.42	0.24	0.73	3280					-4.10	
	Kyrgyzstan	2012	1.67	1.22	2.27	4440	2014	2.27	1.51	3.40	4577		-4.00	0.60		-3.40	
	Macedonia	2011	0.26	0.10	0.67	1376							-5.93				
	Tajikistan	2012	11.58	10.28	13.02	5537						-13.70	-0.15				-13.85
Latin America and the Caribbean	Suriname	2010	1.10	0.73	1.68	3308							-2.29				
	Guyana	2009	12.08	9.96	14.58	2053	2014	11.26	9.75	12.96	3358	3.27	5.33	-0.82	7.77	4.51	8.60
	Belize	2011	4.76	3.81	5.93	1946							-0.86				
	Honduras	2011	6.45	5.87	7.08	10736							-0.01				
	Bolivia											5.64					
	Haiti	2012	20.25	18.57	22.04	7103							1.33				
	Dominican Rep						2014	11.97	11.14	12.85	19981	-3.19			-13.44	-10.25	
Middle East & North Africa	State of Palestine	2010	0.69	0.54	0.88	11106	2014	0.66	0.48	0.92	7816			-0.03			
	Iraq	2011	0.77	0.63	0.93	36307											-1.11
	Yemen						2013	69.30	67.52	71.03	15600					-8.45	
South Asia	India						2015	20.27	19.89	20.65	243867					-38.58	
	Afghanistan	2010	62.66	59.34	65.86	13938	2015	57.70	54.55	60.78	31401			-4.96			
	Nepal	2011	57.74	54.80	60.63	5270	2016	43.79	41.32	46.30	4841		-7.29	-13.95		-21.24	
	Pakistan	2012	66.39	62.81	69.78	11678							-7.03				
	Bangladesh	2012	63.02	61.82	64.21	20903	2014	79.84	78.16	81.43	7799		-27.13	16.82		-10.31	

Table S2: Change in the percentage of children under five whose birth is not registered in 67 countries (1999-2016)

Region	Country	Change in % of children under five whose birth is not registered between first and most recent survey							Percentage of children under five whose birth is not registered					Percentage of children under five whose birth is not registered				
		First survey		Most recent survey		Change			Wave 1 (1999-2004)					Wave 2 (2004-2008)				
		Year	Estimate	Year	Estimate	Years (n)	Total change	Annual change	Year	Estimate	95% CI		Children under 5 (n)	Year	Estimate	95% CI		Children under 5 (n)
	Ghana	2006	48.53	2014	29.30	8	-19.23	-2.40						2006	48.53	44.80	52.3	3464
	Comoros	2000	16.58	2012	12.70	12	-3.88	-0.32	2000	16.58	14.24	19.21	4870					
	Rwanda	2005	17.62	2014	44.01	9	26.39	2.93						2005	17.62	16.23	19.11	8123
	Togo	2000	17.91	2010	22.06	10	4.15	0.42	2000	17.91	15.09	21.12	3129	2006	21.94	19.43	24.68	4073
	Burundi	2000	25.11	2010	24.84	10	-0.27	-0.03	2000	25.11	22.60	27.80	3325	2005	39.79	37.29	42.35	6894
	Zimbabwe	2005	26.06	2015	56.54	10	30.49	3.05						2005	26.06	24.11	28.10	5809
	CAR	2000	27.46	2010	38.97	10	11.51	1.15	2000	27.46	24.95	30.13	14258	2006	50.70	46.90	54.50	9454
	Cote dIvoire	2000	28.16	2011	35.04	11	6.89	0.63	2000	28.16	25.45	31.04	7987	2006	45.06	40.81	49.40	8604
	S Tome & Principe	2000	30.12	2014	4.79	14	-25.33	-1.81	2000	30.12	27.14	33.28	2121	2008	24.94	21.68	28.50	2101
	Namibia	2006	32.90	2013	12.87	7	-20.03	-2.86						2006	32.90	30.79	35.09	5461
	Senegal	2000	34.03	2015	31.74	15	-2.30	-0.15	2000	34.03	30.26	38.02	1316274					
	Burkina Faso	2006	36.34	2010	23.12	4	-13.22	-3.31						2006	36.34	31.94	40.99	5283
	Kenya	2008	39.99	2014	33.12	6	-6.88	-1.15						2008	39.99	36.35	43.75	5956
	Mauritania	2007	44.17	2011	41.16	4	-3.01	-0.75						2007	44.17	41.06	47.32	8658
	Mali	2006	46.73	2012	15.71	6	-31.02	-5.17						2006	46.73	44.00	49.47	13837
Sub-Saharan Africa	Benin	2006	46.93	2011	19.82	5	-27.11	-5.42						2006	46.93	44.80	49.07	16098
	Swaziland	2000	47.05	2014	46.51	14	-0.55	-0.04	2000	47.05	43.35	50.79	3416	2006	70.17	67.69	72.55	3219
	Sierra Leone	2000	53.56	2013	23.29	13	-30.27	-2.33	2000	53.56	49.27	57.80	2710	2008	49.15	46.10	52.20	6709
	Niger	2000	54.49	2012	36.12	12	-18.37	-1.53	2000	54.49	49.38	59.52	5060	2006	68.22	65.00	71.28	9293
	Lesotho	2009	54.86	2014	56.65	5	1.79	0.36										
	Guinea Bissau	2000	57.87	2014	76.34	14	18.47	1.32	2000	57.87	54.02	61.63	5856	2006	61.11	58.30	63.85	5846
	Congo DR	2001	65.83	2013	75.39	12	9.55	0.80	2001	65.83	62.43	69.09	8864	2007	68.72	64.51	72.64	8918
	Cameroon	2000	66.08	2014	33.93	14	-32.15	-2.30	2000	66.08	62.95	69.08	3398	2006	29.90	27.02	32.94	6362
	Gambia	2000	67.78	2013	28.02	13	-39.76	-3.06	2000	67.78	62.84	72.36	3583	2005	44.95	42.05	47.88	6543
	Mozambique	2008	69.16	2011	52.12	3	-17.03	-5.68						2008	69.16	66.35	71.83	11419
	Angola	2001	70.78	2015	74.97	14	4.19	0.30	2001	70.78	68.72	72.76	5282					
	Chad	2000	75.06	2014	87.96	14	12.90	0.92	2000	75.06	70.58	79.06	5384					
	Nigeria	2007	76.69	2013	70.16	6	-6.54	-1.09						2007	76.69	74.35	78.89	15511
	Uganda	2006	78.98	2011	70.12	5	-8.86	-1.77						2006	78.98	76.58	81.20	8399
	Tanzania	2010	83.75	2015	73.61	5	-10.14	-2.03										
	Zambia	1999	90.43	2013	88.71	14	-1.72	-0.12	1999	90.43	88.23	92.25	1563275	2007	85.99	83.85	87.88	6341
	Liberia	2007	96.43	2013	75.38	6	-21.04	-3.51						2007	96.43	95.38	97.24	6028

Table S2: Change in the percentage of children under five whose birth is not registered in 67 countries (1999-2016)

Region	Country	Percentage of children under five whose birth is not registered Wave 3 (2009-2012)					Percentage of children under five whose birth is not registered Wave 4 (2013-2015)					Interval change in percentage of children under five whose birth is not registered (% points)					
		Year	Estimate	95% CI		Children under 5 (n)	Year	Estimate	95% CI		Children under 5 (n)	Wave 1 to 2	Wave 2 to 3	Wave 3 to 4	Wave 1 to 4	Wave 2 to 4	Wave 1 to 3
Sub-Saharan Africa	Ghana	2011	37.51	35.25	39.83	7550	2014	29.30	26.6099	32.15	5496		-11.0205	-8.21		-19.23	
	Comoros	2012	12.70	10.87	14.78	3392											-3.88
	Rwanda	2010	36.76	34.86	38.69	8971	2014	44.01	41.82	46.22	7915		19.14	7.25		26.39	
	Togo	2010	22.06	19.77	24.54	4746						4.04	0.12				4.15
	Burundi	2010	24.84	22.45	27.40	7600						14.68	-14.95				-0.27
	Zimbabwe	2010	51.22	49.00	53.43	5913	2015	56.54	54.33	58.73	6615		25.16	5.33		30.49	
	CAR	2010	38.97	36.56	41.43	10474						23.24	-11.73				11.51
	Cote dIvoire	2011	35.04	32.27	37.92	7773						16.91	-10.02				6.89
	S Tome & Principe						2014	4.79	3.62	6.32	2030	-5.18			-25.33	-20.15	
	Namibia						2013	12.87	11.43	14.47	5678					-20.03	
	Senegal	2010	25.42	23.33	27.62	12227	2015	31.74	28.69	34.96	6280			6.32	-2.30		-8.62
	Burkina Faso	2010	23.12	21.44	24.89	14704							-13.22				
	Kenya						2014	33.12	31.63	34.64	19954					-6.88	
	Mauritania	2011	41.16	39.00	43.35	9278							-3.01				
	Mali	2012	15.71	13.79	17.84	10749							-31.02				
	Benin	2011	19.82	18.34	21.39	14162							-27.11				
	Swaziland	2010	50.54	48.08	53.00	2647	2014	46.51	43.64	49.40	2693	23.12	-19.63	-4.04	-0.55	-23.67	3.49
	Sierra Leone	2010	22.01	19.83	24.35	8583	2013	23.29	20.71	26.08	12282	-4.41	-27.14	1.28	-30.27	-25.86	-31.55
	Niger	2012	36.12	33.46	38.87	13584						13.73	-32.10				-18.37
	Lesotho	2009	54.86	52.50	57.20	4174	2014	56.65	54.02	59.25	3719			1.79			
	Guinea Bissau						2014	76.34	74.25	78.31	7573	3.24			18.47	15.23	
	Congo DR	2010	72.23	68.80	75.42	11093	2013	75.39	72.33	78.21	18937	2.89	3.51	3.16	9.55	6.67	6.40
	Cameroon	2011	38.56	36.21	40.96	11802	2014	33.93	31.35	36.61	7081	-36.18	8.66	-4.63	-32.15	4.03	-27.52
	Gambia						2013	28.02	25.52	30.66	8766	-22.83			-39.76	-16.93	
	Mozambique	2011	52.12	50.06	54.18	10718							-17.03				
	Angola						2015	74.97	73.25	76.62	15189				4.19		
	Chad	2010	84.36	82.94	85.68	17006	2014	87.96	86.73	89.09	18933			3.60	12.90		9.30
	Nigeria	2011	58.50	56.48	60.49	25192	2013	70.16	68.25	71.99	30109		-18.20	11.66		-6.54	
	Uganda	2011	70.12	67.57	72.56	8361							-8.86				
	Tanzania	2010	83.75	82.26	85.14	8081	2015	73.61	71.88	75.27	10091			-10.14			
	Zambia						2013	88.71	87.35	89.93	13716	-4.44			-1.72	2.72	
	Liberia						2013	75.38	72.70	77.88	7344					-21.04	

Notes: Estimates presented should be consistent with published DHS and MICS reports. Estimates in the following surveys are different from the reports: Benin (DHS, 2006), Indonesia (DHS 2007), Mongolia (MICS 2000), Senegal (DHS, 2015). Sample sizes of children under five in the following surveys are different rom the reports: Kazakhstan (MICS 2015), Nigeria (MICS 2007), Swaziland (MICS 2000).

Table S3: Change in wealth inequalities in the percentage of children under five whose birth is not registered in 67 countries (1999-2016)

Table S3: Change in wealth inequities in the percentage of children under five whose birth is not registered in 67 countries (1999-2016)

Region	Country	Total change in SII between first and most recent survey							% of children under five whose birth is not registered by wealth quintile and SII						% of children under five whose birth is not registered by wealth quintile and SII					
		First survey		Most recent survey		Change			Wave 1 (1999-2004)						Wave 2 (2004-2008)					
									Year	Average (%)		SII			Year	Average (%)		SII		
		Year	Estimate	Year	Estimate	Years (n)	Total change	Annual change		Q1&2	Q3-5	Estimate	95% CI			Q1&2	Q3-5	Estimate	95% CI	
East Asia & Pacific	Cambodia	2005	20.70	2014	34.24	9	13.55	1.51							2005	38.40	28.94	20.70	13.88	27.51
	Indonesia	2007	64.03	2012	56.66	5	-7.37	-1.47							2007	68.07	35.62	64.03	60.44	67.63
	Lao	2000	0.88	2011	30.65	11	29.77	2.71	2000	41.33	39.97	0.88	-9.74	11.49	2006	36.25	19.59	30.51	22.64	38.38
	Mongolia	2000	1.34	2013	0.61	13	-0.73	-0.06	2000	2.60	2.21	1.34	-0.15	2.82	2005	1.45	1.85	0.85	-0.55	2.25
	Myanmar	2000	39.10	2015	37.42	15	-1.67	-0.11	2000	49.40	30.08	39.10	33.23	44.96						
	Thailand	2005	1.06	2012	1.54	7	0.48	0.07							2005	0.95	0.37	1.06	0.27	1.85
	Vietnam	2000	56.31	2013	9.56	13	-46.75	-3.60	2000	41.11	13.40	56.31	47.87	64.75	2006	22.76	5.93	29.95	22.81	37.09
Europe & Central Asia	Albania	2000	0.07	2008	1.29	8	1.22	0.15	2000	1.28	1.09	0.07	-2.69	2.83	2008	1.60	1.16	1.29	-0.90	3.48
	Armenia	2005	8.71	2010	0.73	5	-7.99	-1.60							2005	6.59	1.72	8.71	1.35	16.08
	Azerbaijan	2000	3.19	2006	6.19	6	3.01	0.50	2000	4.56	1.82	3.19	-0.20	6.57	2006	8.28	4.79	6.19	1.15	11.24
	Kazakhstan	2006	0.49	2015	0.49	9	0.00	0.00							2006	0.86	0.69	0.49	-0.61	1.60
	Kyrgyzstan	2005	1.20	2014	3.51	9	2.32	0.26							2005	5.98	5.48	1.20	-2.93	5.32
	Macedonia	2005	12.60	2011	0.89	6	-11.71	-1.95							2005	9.34	3.04	12.60	1.68	23.53
	Moldova	2000	1.06	2012	0.88	12	-0.18	-0.01	2000	2.34	1.97	1.06	-1.96	4.08	2005	6.51	8.41	2.53	-2.55	7.60
	Montenegro	2005	5.53	2013	0.60	8	-4.93	-0.62							2005	3.84	0.71	5.53	-2.88	13.95
	Serbia	2005	1.64	2014	1.74	9	0.10	0.01							2005	1.60	0.71	1.64	-0.13	3.41
	Tajikistan	2000	3.48	2012	5.65	12	2.17	0.18	2000	26.44	24.69	3.48	-5.61	12.56	2005	12.80	10.99	2.63	-4.10	9.37
	Turkmenistan	2006	3.12	2015	0.43	9	-2.69	-0.30							2006	5.23	3.98	3.12	-0.78	7.02
	Ukraine	2005	0.49	2012	0.83	7	0.35	0.05							2005	0.04	0.29	0.49	-0.22	1.19
	Uzbekistan	2000	0.19	2006	0.01	6	-0.17	-0.03	2000	0.59	0.36	0.19	-0.73	1.10	2006	0.05	0.13	0.01	-0.20	0.23
Latin America and the Caribbean	Belize	2006	6.51	2011	1.50	5	-5.01	-1.00							2006	8.17	3.42	6.51	0.54	12.48
	Bolivia	2000	13.97	2008	26.08	8	12.11	1.51	2000	22.53	15.18	13.97	8.06	19.88	2008	30.46	18.65	26.08	21.86	30.30
	Dominican Rep	2000	50.79	2014	32.32	14	-18.47	-1.32	2000	40.93	11.91	50.79	42.71	58.87	2007	34.61	11.03	43.68	39.62	47.74
	Guyana	2000	8.60	2014	13.79	14	5.19	0.37	2000	5.28	1.73	8.60	5.09	12.10	2006	9.26	4.03	13.00	7.39	18.61
	Haiti	2005	25.07	2012	23.74	7	-1.33	-0.19							2005	25.50	13.41	25.07	18.74	31.40
	Honduras	2005	4.17	2011	4.12	6	-0.05	-0.01							2005	7.31	5.73	4.17	2.17	6.17
	Suriname	2006	6.95	2010	3.08	4	-3.87	-0.97							2006	4.88	1.69	6.95	2.09	11.81
Middle East & North Africa	Iraq	2000	2.32	2011	1.96	11	-0.37	-0.03	2000	2.40	1.42	2.32	1.22	3.43						
	State of Palestine	2010	0.32	2014	0.63	4	0.30	0.08												
	Yemen	2006	52.77	2013	44.39	7	-8.38	-1.20							2006	92.02	66.26	52.77	41.28	64.26
South Asia	Afghanistan	2010	25.77	2015	43.94	5	18.17	3.63												
	Bangladesh	2006	13.78	2014	14.15	8	0.37	0.05							2006	93.57	87.21	13.78	11.69	15.88
	India	2005	53.26	2015	35.34	10	-17.92	-1.79							2005	72.77	46.38	53.26	49.91	56.61
	Nepal	2006	29.09	2016	5.59	10	-23.50	-2.35							2006	73.29	57.93	29.09	22.05	36.13
	Pakistan	2006	25.29	2012	71.89	6	46.60	7.77							2006	81.44	66.94	25.29	18.82	31.77

Table S3: Change in wealth inequities in the percentage of children under five whose birth is not registered in 67 countries (1999-2016)

Region	Country	% of children under five whose birth is not registered by wealth quintile and SII						% of children under five whose birth is not registered by wealth quintile and SII						Interval change in SII (% points) *[SII based on 5 wealth quintiles]*					
		Wave 3 (2009-2012)						Wave 4 (2013-2015)											
		Year	Average (%)		SII			Year	Average (%)		SII								
			Q1&2	Q3-5	Estimate	95% CI			Q1&2	Q3-5	Estimate	95% CI		Wave 1 to 2	Wave 2 to 3	Wave 3 to 4	Wave 1 to 4	Wave 2 to 4	Wave 1 to 3
East Asia & Pacific	Cambodia	2010	46.41	30.23	33.98	28.55	39.41	2014	36.09	19.18	34.24	27.74	40.75		13.28	0.26		13.55	
	Indonesia	2012	50.50	21.06	56.66	52.96	60.37								-7.37				
	Lao	2011	33.02	17.33	30.65	24.79	36.51							29.63	0.14				29.77
	Mongolia	2010	0.99	1.07	0.24	-1.02	1.49	2013	0.82	0.62	0.61	-0.13	1.34	-0.49	-0.61	0.37	-0.73	-0.24	-1.10
	Myanmar							2015	28.33	8.82	37.42	28.58	46.27				-1.67		
	Thailand	2012	1.04	0.09	1.54	0.61	2.48								0.48				
	Vietnam	2010	8.99	2.29	14.01	7.89	20.13	2013	6.74	2.00	9.56	5.61	13.51	-26.36	-15.94	-4.45	-46.75	-20.39	-42.30
Europe & Central Asia	Albania													1.22					
	Armenia	2010	0.00	0.70	0.73	-0.20	1.66								-7.99				
	Azerbaijan													3.01					
	Kazakhstan	2010	0.34	0.18	0.49	0.04	0.94	2015	0.43	0.25	0.49	-0.27	1.25		0.00	0.00		0.00	
	Kyrgyzstan	2012	1.30	1.93	0.99	-0.56	2.54	2014	3.17	1.55	3.51	-0.80	7.82		-0.20	2.52		2.32	
	Macedonia	2011	0.49	0.08	0.89	-0.15	1.93								-11.71				
	Moldova	2012	0.76	0.22	0.88	-0.24	2.00							1.47	-1.65				-0.18
	Montenegro							2013	0.92	0.40	0.60	-1.51	2.71					-4.93	
	Serbia	2010	1.87	0.59	2.83	-1.75	7.41	2014	1.42	0.21	1.74	-0.46	3.95		1.19	-1.09		0.10	
	Tajikistan	2012	13.57	10.20	5.65	1.37	9.93							-0.84	3.01				2.17
	Turkmenistan							2015	0.48	0.38	0.43	-0.33	1.18					-2.69	
	Ukraine	2012	0.04	0.32	0.83	-0.56	2.22								0.35				
	Uzbekistan													-0.17					
Latin America and the Caribbean	Belize	2011	5.16	4.38	1.50	-2.39	5.38								-5.01				
	Bolivia													12.11					
	Dominican Rep							2014	20.21	4.87	32.32	29.38	35.26	-7.11			-18.47	-11.36	
	Guyana	2009	13.37	10.78	7.61	-0.47	15.69	2014	14.52	7.68	13.79	8.41	19.16	4.40	-5.39	6.18	5.19	0.79	-0.99
	Haiti	2012	26.53	14.74	23.74	18.44	29.04								-1.33				
	Honduras	2011	7.31	5.73	4.12	2.05	6.18								-0.05				
	Suriname	2010	1.74	0.34	3.08	1.82	4.35								-3.87				
Middle East & North Africa	Iraq	2011	1.32	0.29	1.96	1.40	2.51												-0.37
	State of Palestine	2010	0.79	0.60	0.32	-0.30	0.94	2014	0.36	0.92	0.63	-0.08	1.33			0.30			
	Yemen							2013	81.59	59.78	44.39	39.50	49.28					-8.38	
South Asia	Afghanistan	2010	67.60	59.01	25.77	17.63	33.91	2015	69.59	49.89	43.94	37.05	50.83			18.17			
	Bangladesh	2012	67.61	59.29	17.57	13.81	21.33	2014	83.07	77.56	14.15	9.64	18.67		3.79	-3.42		0.37	
	India							2015	29.90	11.84	35.34	34.19	36.49					-17.92	
	Nepal	2011	61.47	54.31	16.23	7.06	25.40	2016	41.63	45.38	5.59	-2.65	13.84		-12.86	-10.64		-23.50	
	Pakistan	2012	88.58	48.26	71.89	67.38	76.41								46.60				

Table S3: Change in wealth inequities in the percentage of children under five whose birth is not registered in 67 countries (1999-2016)

Region	Country	Total change in SII between first and most recent survey							% of children under five whose birth is not registered by wealth quintile and SII						% of children under five whose birth is not registered by wealth quintile and SII					
		First survey		Most recent survey		Change			Wave 1 (1999-2004)						Wave 2 (2004-2008)					
									Year	Average (%)		SII			Year	Average (%)		SII		
		Year	Estimate	Year	Estimate	Years (n)	Total change	Annual change		Q1&2	Q3-5	Estimate	95% CI			Q1&2	Q3-5	Estimate	95% CI	
Sub-Saharan Africa	Angola	2001	35.31	2015	44.82	14	9.51	0.68	2001	80.89	65.27	35.31	29.80	40.82						
	Benin	2006	48.44	2011	42.13	5	-6.31	-1.26							2006	60.50	36.67	48.44	43.87	53.00
	Burkina Faso	2006	42.32	2010	38.85	4	-3.47	-0.87							2006	47.74	28.14	42.32	32.90	51.75
	Burundi	2000	9.32	2010	25.30	10	15.98	1.60	2000	29.24	22.72	9.32	2.45	16.18	2005	41.63	38.51	7.25	0.87	13.64
	CAR	2000	47.01	2010	45.66	10	-1.35	-0.13	2000	42.26	16.71	47.01	42.28	51.73	2006	70.73	36.08	66.16	61.41	70.90
	Cameroon	2000	6.85	2014	64.27	14	57.42	4.10	2000	69.43	63.49	6.85	-3.05	16.75	2006	44.58	17.55	50.08	43.03	57.12
	Chad	2000	35.25	2014	30.26	14	-4.99	-0.36	2000	83.11	68.61	35.25	25.08	45.42						
	Comoros	2000	23.21	2012	9.62	12	-13.60	-1.13	2000	22.79	12.38	23.21	16.25	30.18						
	Congo DR	2001	6.54	2013	22.22	12	15.68	1.31	2001	64.11	66.94	6.54	-2.68	15.77	2007	70.23	67.60	5.92	-3.75	15.59
	Cote dIvoire	2000	47.16	2011	51.76	11	4.60	0.42	2000	40.68	17.00	47.16	41.61	52.71	2006	65.27	27.58	69.45	63.98	74.91
	Gambia	2000	12.66	2013	4.06	13	-8.60	-0.66	2000	68.94	67.03	12.66	1.21	24.10	2005	44.82	45.05	7.12	-0.60	14.84
	Ghana	2006	57.16	2014	38.10	8	-19.06	-2.38							2006	64.89	34.30	57.16	50.40	63.91
	Guinea Bissau	2000	39.33	2014	33.43	14	-5.90	-0.42	2000	44.43	67.64	39.33	33.07	45.59	2006	73.34	51.34	43.23	37.42	49.04
	Kenya	2008	35.36	2014	43.02	6	7.66	1.28							2008	49.32	32.06	35.36	24.16	46.56
	Lesotho	2009	9.66	2014	30.36	5	20.70	4.14												
	Liberia	2007	6.57	2013	17.54	6	10.97	1.83							2007	98.18	94.88	6.57	3.34	9.80
	Mali	2006	41.25	2012	29.64	6	-11.61	-1.93							2006	56.67	39.97	41.25	33.56	48.94
	Mauritania	2007	68.46	2011	61.91	4	-6.55	-1.64							2007	65.65	26.65	68.46	63.85	73.07
	Mozambique	2008	32.59	2011	18.94	3	-13.65	-4.55							2008	78.44	61.67	32.59	26.14	39.04
	Namibia	2006	50.95	2013	12.27	7	-38.68	-5.53							2006	46.39	21.37	50.95	45.41	56.49
	Niger	2000	45.58	2012	37.09	12	-8.49	-0.71	2000	65.59	47.50	45.58	37.01	54.15	2006	79.63	60.03	47.22	40.00	54.44
	Nigeria	2007	52.24	2013	65.38	6	13.14	2.19							2007	90.85	67.31	52.24	45.70	58.79
	Rwanda	2005	2.42	2014	28.41	9	25.99	2.89							2005	17.08	18.00	2.42	-1.63	6.47
	S Tome & Principe	2000	29.34	2014	16.22	14	-13.12	-0.94	2000	37.69	24.57	29.34	21.77	36.91	2008	27.12	23.48	10.85	1.23	20.46
	Senegal	2000	57.67	2015	57.78	15	0.11	0.01	2000	52.36	20.77	57.67	51.27	64.07						
	Sierra Leone	2000	52.47	2013	4.71	13	-47.76	-3.67	2000	68.64	42.02	52.47	44.35	60.58	2008	55.19	44.45	21.87	13.91	29.82
	Swaziland	2000	29.31	2014	41.15	14	11.84	0.85	2000	54.17	39.51	29.31	19.94	38.69	2006	78.76	62.68	33.27	26.02	40.52
	Tanzania	2010	47.00	2015	58.20	5	11.20	2.24												
	Togo	2000	25.75	2010	49.76	10	24.01	2.40	2000	23.88	12.88	25.75	17.75	33.75	2006	36.72	10.90	49.73	43.08	56.38
	Uganda	2006	6.92	2011	16.12	5	9.20	1.84							2006	79.77	78.34	6.92	1.08	12.76
	Zambia	1999	17.19	2013	25.85	14	8.66	0.62	1999	93.76	88.14	17.19	10.54	23.84	2007	93.14	79.77	28.85	21.33	36.38
	Zimbabwe	2005	20.54	2015	58.47	10	37.94	3.79							2005	31.17	21.66	20.54	14.19	26.88

Table S3: Change in wealth inequities in the percentage of children under five whose birth is not registered in 67 countries (1999-2016)

Region	Country	% of children under five whose birth is not registered by wealth quintile and SII						% of children under five whose birth is not registered by wealth quintile and SII						Interval change in SII (% points) *[SII based on 5 wealth quintiles]*					
		Wave 3 (2009-2012)						Wave 4 (2013-2015)											
		Year	Average (%)		SII			Year	Average (%)		SII								
			Q1&2	Q3-5	Estimate	95% CI			Q1&2	Q3-5	Estimate	95% CI		Wave 1 to 2	Wave 2 to 3	Wave 3 to 4	Wave 1 to 4	Wave 2 to 4	Wave 1 to 3
Sub-Saharan Africa	Angola							2015	86.89	64.64	44.82	39.68	49.96				9.51		
	Benin	2011	32.47	10.49	42.13	37.25	47.01								-6.31				
	Burkina Faso	2010	34.56	14.83	38.85	33.93	43.77								-3.47				
	Burundi	2010	31.81	20.04	25.30	20.13	30.48							-2.06	18.05				15.98
	CAR	2010	51.46	29.43	45.66	39.34	51.97							19.15	-20.50				-1.35
	Cameroon	2011	59.38	21.62	68.55	63.85	73.25	2014	52.87	18.12	64.27	57.76	70.78	43.23	18.47	-4.28	57.42	14.19	61.70
	Chad	2010	93.66	78.19	42.36	35.92	48.79	2014	93.80	83.59	30.26	24.78	35.74			-12.09	-4.99		7.11
	Comoros	2012	16.08	9.76	9.62	3.17	16.07												-13.60
	Congo DR	2010	73.47	71.31	3.90	-5.52	13.32	2013	80.53	71.33	22.22	14.71	29.73	-0.62	-2.02	18.32	15.68	16.30	-2.64
	Cote dIvoire	2011	48.57	23.82	51.76	45.89	57.63							22.29	-17.69				4.60
	Gambia							2013	28.85	27.37	4.06	-3.00	11.12	-5.54			-8.60	-3.06	
	Ghana	2011	49.64	28.20	42.76	36.49	49.04	2014	40.38	20.84	38.10	28.65	47.55		-14.40	-4.66		-19.06	
	Guinea Bissau							2014	84.53	69.42	33.43	27.70	39.16	3.90			-5.90	-9.80	
	Kenya							2014	44.90	23.03	43.02	39.12	46.93					7.66	
	Lesotho	2009	57.73	52.48	9.66	2.03	17.29	2014	64.54	50.25	30.36	22.62	38.09			20.70			
	Liberia							2013	80.20	71.20	17.54	10.08	25.00					10.97	
	Mali	2012	23.44	10.15	29.64	24.14	35.13								-11.61				
	Mauritania	2011	60.04	25.44	61.91	56.81	67.01								-6.55				
	Mozambique	2011	57.34	47.93	18.94	12.22	25.66								-13.65				
	Namibia							2013	16.25	9.76	12.27	6.54	18.00					-38.68	
	Niger	2012	45.94	29.33	37.09	30.43	43.75							1.64	-10.13				-8.49
	Nigeria	2011	82.65	39.73	74.50	71.68	77.31	2013	88.35	54.98	65.38	61.97	68.79		22.26	-9.12		13.14	
	Rwanda	2010	39.71	34.37	8.90	3.79	14.00	2014	52.38	36.87	28.41	22.74	34.09		6.48	19.52		25.99	
	S Tome & Principe							2014	9.02	1.60	16.22	10.42	22.01	-18.49			-13.12	5.37	
	Senegal	2010	39.15	13.56	50.72	44.71	56.72	2015	48.26	17.58	57.78	49.84	65.71			7.06	0.11		-6.95
	Sierra Leone	2010	26.08	18.67	15.19	7.46	22.91	2013	24.42	22.35	4.71	-1.53	10.95	-30.60	-6.68	-10.48	-47.76	-17.16	-37.28
	Swaziland	2010	59.65	42.96	36.77	29.95	43.60	2014	57.21	37.00	41.15	32.44	49.86	3.96	3.51	4.38	11.84	7.88	7.46
	Tanzania	2010	94.77	74.74	47.00	42.06	51.93	2015	88.81	60.71	58.20	53.72	62.68			11.20			
	Togo	2010	35.49	10.86	49.76	43.03	56.49							23.99	0.03				24.01
	Uganda	2011	73.53	67.48	16.12	7.52	24.72								9.20				
	Zambia							2013	94.90	83.21	25.85	20.79	30.91	11.6614			8.66	-3.00	
	Zimbabwe	2010	61.86	42.46	40.48	34.31	46.65	2015	72.69	43.47	58.47	53.74	63.21		19.9434	18.00		37.9388	

Table S4: Change in urban/rural inequalities in the percentage of children under five whose birth is not registered in 67 countries (1999-2016)

Table S4: Change in urban/rural inequities in the percentage of children under five whose birth is not registered in 67 countries (1999-2016)

Region	Country	Total change in urban/rural difference between first and most recent survey							% of children under five whose birth is not registered by iurban/rural residience and urban/rural difference						% of children under five whose birth is not registered by iurban/rural residience and urban/rural difference					
		First survey		Most recent survey			Change		Wave 1 (1999-2004)						Wave 2 (2004-2008)					
										Average (%)		Difference (% points)				Average (%)		Difference (% points)		
		Year	Estimate	Year	Estimate	Years (n)	Total change	Annual change	Year	Rural	Urban	Estimate	95% CI		Year	Rural	Urban	Estimate	95% CI	
East Asia & Pacific	Cambodia	2005	5.32	2014	12.81	9	7.49	0.83							2005	34.34	29.02	5.32	-0.93	11.57
	Indonesia	2007	26.29	2012	17.98	5	-8.30	-1.66							2007	60.20	33.91	26.29	22.73	29.85
	Lao	2000	15.41	2011	16.48	11	1.07	0.10	2000	44.27	28.86	15.41	7.82	23.00	2006	31.04	16.07	14.97	9.93	20.01
	Mongolia	2000	0.65	2013	0.33	13	-0.32	-0.02	2000	2.66	2.01	0.65	-0.26	1.55	2005	1.35	1.96	-0.61	-1.52	0.30
	Myanmar	2000	33.82	2015	16.20	15	-17.63	-1.18	2000	46.66	12.84	33.82	28.79	38.85						
	Thailand	2005	0.28	2012	-0.60	7	-0.88	-0.13							2005	0.70	0.42	0.28	-0.19	0.74
	Vietnam	2000	23.68	2013	0.98	13	-22.71	-1.75	2000	32.22	8.53	23.68	17.38	29.99	2006	14.28	5.73	8.55	4.55	12.55
Europe & Central Asia	Albania	2000	-0.17	2008	0.58	8	0.76	0.09	2000	1.11	1.28	-0.17	-1.45	1.11	2008	1.58	1.00	0.58	-0.70	1.86
	Armenia	2005	1.88	2010		5									2005	4.76	2.88	1.88	-1.97	5.74
	Azerbaijan	2000	2.61	2006	3.84	6	1.23	0.21	2000	4.47	1.86	2.61	0.33	4.90	2006	8.30	4.45	3.84	0.34	7.35
	Kazakhstan	2006	0.03	2015	0.36	9	0.33	0.04							2006	0.79	0.76	0.03	-0.63	0.68
	Kyrgyzstan	2005	2.79	2014	1.11	9	-1.68	-0.19							2005	6.80	4.01	2.79	-1.27	6.85
	Macedonia	2005	1.66	2011	0.37	6	-1.30	-0.22							2005	7.09	5.43	1.66	-3.91	7.24
	Moldova	2000	0.23	2012	0.25	12	0.02	0.00	2000	2.22	1.99	0.23	-1.34	1.80	2005	7.47	7.98	-0.52	-3.59	2.56
	Montenegro	2005	-0.85	2013	-0.48	8	0.37	0.05							2005	1.52	2.37	-0.85	-4.29	2.59
	Serbia	2005	0.26	2014	0.28	9	0.02	0.00							2005	1.19	0.92	0.26	-0.61	1.14
	Tajikistan	2000	3.03	2012	-0.81	12	-3.83	-0.32	2000	26.06	23.04	3.03	-4.37	10.43	2005	10.52	15.12	-4.60	-8.68	-0.52
	Turkmenistan	2006	0.53	2015	-0.30	9	-0.84	-0.09							2006	4.71	4.17	0.53	-1.87	2.93
	Ukraine	2005	-0.22	2012	-0.27	7	-0.04	-0.01							2005	0.04	0.27	-0.22	-0.56	0.12
	Uzbekistan	2000	-0.03	2006	0.08	6	0.11	0.02	2000	0.47	0.50	-0.03	-0.57	0.50	2006	0.12	0.04	0.08	-0.08	0.24
Latin America and the Caribbean	Belize	2006	-4.63	2011	-0.98	5	3.65	0.73							2006	3.53	8.16	-4.63	-8.74	-0.53
	Bolivia	2000	3.83	2008	7.13	8	3.29	0.41	2000	20.78	16.95	3.83	0.00	7.66	2008	27.99	20.87	7.13	4.49	9.76
	Dominican Rep	2000	16.87	2014	7.83	14	-9.03	-0.65	2000	34.54	17.67	16.87	10.43	23.30	2007	28.94	18.96	9.98	6.96	13.01
	Guyana	2000	2.85	2014	2.31	14	-0.54	-0.04	2000	4.26	1.41	2.85	0.88	4.82	2006	7.62	4.11	3.51	0.71	6.32
	Haiti	2005	8.20	2012	7.84	7	-0.36	-0.05							2005	21.66	13.47	8.20	4.89	11.50
	Honduras	2005	2.11	2011	1.96	6	-0.15	-0.03							2005	7.34	5.23	2.11	0.96	3.26
	Suriname	2006	2.56	2010	1.78	4	-0.78	-0.20							2006	4.97	2.41	2.56	0.10	5.01
Middle East & North Africa	Iraq	2000	1.51	2011	0.49	11	-1.01	-0.09	2000	2.84	1.33	1.51	0.88	2.14						
	State of Palestine	2010	-0.09	2014	-0.26	4	-0.17	-0.04												
	Yemen	2006	21.77	2013	24.08	7	2.31	0.33							2006	83.62	61.85	21.77	13.62	29.92
South Asia	Afghanistan	2010	27.00	2015	27.53	5	0.52	0.10												
	Bangladesh	2006	3.69	2014	3.50	8	-0.19	-0.02							2006	91.15	87.46	3.69	2.08	5.30
	India	2005	24.53	2015	12.74	10	-11.79	-1.18							2005	65.19	40.66	24.53	21.76	27.31
	Nepal	2006	7.92	2016	-3.00	10	-10.92	-1.09							2006	66.03	58.11	7.92	1.13	14.70
	Pakistan	2006	8.06	2012	36.45	6	28.38	4.73							2006	75.84	67.77	8.06	3.37	12.76

Table S4: Change in urban/rural inequities in the percentage of children under five whose birth is not registered in 67 countries (1999-2016)

Region	Country	% of children under five whose birth is not registered by iurban/rural residience and urban/rural difference						% of children under five whose birth is not registered by iurban/rural residence and urban/rural difference						Interval change in urban/rural difference (% points)					
		Wave 3 (2009-2012)						Wave 4 (2013-2015)											
		Year	Average (%)		Difference (% points)			Year	Average (%)		Difference (% points)								
			Rural	Urban	Estimate	95% CI			Rural	Urban	Estimate	95% CI		Wave 1 to 2	Wave 2 to 3	Wave 3 to 4	Wave 1 to 4	Wave 2 to 4	Wave 1 to 3
East Asia & Pacific	Cambodia	2010	40.12	25.59	14.53	10.40	18.67	2014	28.44	15.63	12.81	9.34	16.28		9.21	-1.72		7.49	
	Indonesia	2012	42.19	24.20	17.98	15.00	20.97								-8.30				
	Lao	2011	28.69	12.21	16.48	13.17	19.80							-0.44	1.52				1.07
	Mongolia	2010	0.90	1.13	-0.23	-0.92	0.46	2013	0.90	0.57	0.33	-0.11	0.77	-1.26	0.38	0.56	-0.32	0.94	-0.87
	Myanmar							2015	22.31	6.11	16.20	12.32	20.07				-17.63		
	Thailand	2012	0.25	0.85	-0.60	-1.11	-0.09								-0.88				
	Vietnam	2010	5.83	2.93	2.89	0.79	4.99	2013	4.23	3.25	0.98	-0.57	2.52	-15.13	-5.66	-1.92	-22.71	-7.58	-20.79
Europe & Central Asia	Albania													0.76					
	Armenia	2010	0.00	0.71															
	Azerbaijan													1.23					
	Kazakhstan	2010	0.38	0.13	0.25	-0.01	0.51	2015	0.50	0.14	0.36	0.00	0.72		0.22	0.11		0.33	
	Kyrgyzstan	2012	1.87	1.12	0.75	-0.21	1.72	2014	2.60	1.49	1.11	-0.36	2.58		-2.04	0.36		-1.68	
	Macedonia	2011	0.44	0.08	0.37	-0.13	0.86								-1.30				
	Moldova	2012	0.52	0.27	0.25	-0.31	0.81							-0.74	0.77				0.02
	Montenegro							2013	0.29	0.76	-0.48	-1.37	0.42					0.37	
	Serbia	2010	0.75	1.36	-0.62	-2.40	1.17	2014	0.76	0.48	0.28	-0.69	1.24		-0.88	0.89		0.02	
	Tajikistan	2012	11.41	12.21	-0.81	-3.82	2.21							-7.63	3.80				-3.83
	Turkmenistan							2015	0.31	0.62	-0.30	-0.81	0.20					-0.84	
	Ukraine	2012	0.02	0.29	-0.27	-0.69	0.15								-0.04				
	Uzbekistan													0.11					
Latin America and the Caribbean	Belize	2011	4.38	5.37	-0.98	-3.19	1.22								3.65				
	Bolivia													3.29					
	Dominican Rep							2014	17.82	9.99	7.83	5.67	10.00	-6.88			-9.03	-2.15	
	Guyana	2009	13.01	8.90	4.11	-0.32	8.54	2014	11.83	9.53	2.31	-1.02	5.63	0.66	0.60	-1.80	-0.54	-1.20	1.26
	Haiti	2012	22.87	15.03	7.84	4.71	10.97								-0.36				
	Honduras	2011	7.33	5.37	1.96	0.74	3.18								-0.15				
	Suriname	2010	2.18	0.40	1.78	0.70	2.85								-0.78				
Middle East & North Africa	Iraq	2011	1.10	0.60	0.49	0.19	0.80												-1.01
	State of Palestine	2010	0.62	0.70	-0.09	-0.59	0.42	2014	0.45	0.70	-0.26	-0.76	0.25			-0.17			
	Yemen							2013	75.89	51.81	24.08	20.40	27.76					2.31	
South Asia	Afghanistan	2010	67.00	40.00	27.00	21.57	32.44	2015	64.03	36.50	27.53	21.21	33.85			0.52			
	Bangladesh	2012	64.54	57.14	7.40	4.00	10.80	2014	80.74	77.24	3.50	0.21	6.79		3.71	-3.90		-0.19	
	India							2015	23.90	11.16	12.74	12.01	13.48					-11.79	
	Nepal	2011	57.94	55.81	2.14	-2.91	7.18	2016	42.17	45.17	-3.00	-7.98	1.97		-5.78	-5.14		-10.92	
	Pakistan	2012	77.19	40.74	36.45	30.00	42.89								28.38				

Table S4: Change in urban/rural inequities in the percentage of children under five whose birth is not registered in 67 countries (1999-2016)

Region	Country	Total change in urban/rural difference between first and most recent survey							% of children under five whose birth is not registered by iurban/rural residience and urban/rural difference						% of children under five whose birth is not registered by iurban/rural residience and urban/rural difference					
		First survey		Most recent survey			Change		Wave 1 (1999-2004)						Wave 2 (2004-2008)					
									Year	Average (%)		Difference (% points)			Year	Average (%)		Difference (% points)		
		Year	Estimate	Year	Estimate	Years (n)	Total change	Annual change		Rural	Urban	Estimate	95% CI			Rural	Urban	Estimate	95% CI	
Sub-Saharan Africa	Angola	2001	14.44	2015	19.26	14	4.82	0.34	2001	80.77	66.33	14.44	10.72	18.16						
	Benin	2006	14.56	2011	11.10	5	-3.46	-0.69							2006	51.94	37.38	14.56	10.30	18.82
	Burkina Faso	2006	27.48	2010	19.31	4	-8.17	-2.04							2006	41.61	14.13	27.48	19.23	35.73
	Burundi	2000	-3.99	2010	12.45	10	16.44	1.64	2000	24.82	28.81	-3.99	-12.99	5.02	2005	39.89	37.82	2.07	-5.09	9.23
	CAR	2000	24.42	2010	26.73	10	2.31	0.23	2000	36.97	12.55	24.42	20.82	28.02	2006	64.19	27.83	36.36	31.80	40.91
	Cameroon	2000	-4.10	2014	31.12	14	35.22	2.52	2000	64.88	68.98	-4.10	-9.96	1.76	2006	42.36	14.32	28.04	23.39	32.69
	Chad	2000	35.06	2014	29.23	14	-5.83	-0.42	2000	82.51	47.44	35.06	28.35	41.77						
	Comoros	2000	4.38	2012	3.07	12	-1.31	-0.11	2000	17.49	13.11	4.38	-0.52	9.28						
	Congo DR	2001	-6.44	2013	7.75	12	14.18	1.18	2001	63.77	70.21	-6.44	-13.08	0.21	2007	69.58	67.44	2.14	-5.99	10.27
	Cote dIvoire	2000	28.09	2011	30.94	11	2.85	0.26	2000	40.09	12.00	28.09	24.24	31.94	2006	59.47	20.75	38.72	32.97	44.48
	Gambia	2000	7.65	2013	-0.48	13	-8.13	-0.63	2000	70.67	63.02	7.65	-0.88	16.17	2005	46.07	42.88	3.19	-2.05	8.44
	Ghana	2006	26.42	2014	15.46	8	-10.96	-1.37							2006	57.97	31.54	26.42	20.27	32.58
	Guinea Bissau	2000	-14.97	2014	16.91	14	31.89	2.28	2000	53.35	68.33	-14.97	-20.33	-9.62	2006	66.95	46.92	20.03	15.03	25.04
	Kenya	2008	19.58	2014	17.80	6	-1.78	-0.30							2008	43.27	23.69	19.58	12.42	26.73
	Lesotho	2009	-2.15	2014	13.74	5	15.89	3.18												
	Liberia	2007	2.45	2013	9.06	6	6.61	1.10							2007	97.17	94.72	2.45	0.21	4.70
	Mali	2006	30.08	2012	13.66	6	-16.43	-2.74							2006	55.17	25.09	30.08	21.85	38.32
	Mauritania	2007	32.78	2011	26.54	4	-6.24	-1.56							2007	57.63	24.85	32.78	27.93	37.63
	Mozambique	2008	10.76	2011	3.75	3	-7.01	-2.34							2008	72.21	61.45	10.76	5.91	15.60
	Namibia	2006	23.17	2013	2.99	7	-20.18	-2.88							2006	40.66	17.49	23.17	19.31	27.03
	Niger	2000	45.00	2012	32.04	12	-12.96	-1.08	2000	60.41	15.41	45.00	38.50	51.51	2006	75.29	29.35	45.95	40.27	51.63
	Nigeria	2007	27.70	2013	31.25	6	3.56	0.59							2007	85.06	57.36	27.70	22.89	32.50
	Rwanda	2005	-4.42	2014	-0.67	9	3.74	0.42							2005	16.98	21.40	-4.42	-8.04	-0.80
	S Tome & Principe	2000	6.08	2014	3.39	14	-2.69	-0.19	2000	32.92	26.84	6.08	0.00	12.17	2008	26.21	23.62	2.58	-4.16	9.32
	Senegal	2000	32.64	2015	29.22	15	-3.42	-0.23	2000	45.14	12.50	32.64	27.49	37.80						
	Sierra Leone	2000	25.53	2013	3.84	13	-21.70	-1.67	2000	59.87	34.34	25.53	16.57	34.50	2008	52.19	40.87	11.32	5.23	17.42
	Swaziland	2000	22.90	2014	12.94	14	-9.96	-0.71	2000	50.73	27.83	22.90	14.65	31.15	2006	71.90	61.89	10.00	4.00	16.01
	Tanzania	2010	34.57	2015	33.23	5	-1.34	-0.27												
	Togo	2000	15.17	2010	22.71	10	7.54	0.753778	2000	22.04	6.87	15.17	10.30	20.04	2006	30.69	7.21	23.48	19.76	27.20
	Uganda	2006	2.95	2011	9.34	5	6.39	1.28							2006	79.30	76.36	2.95	-3.42	9.31
	Zambia	1999	-9.10	2013	13.67	14	22.78	1.63	1999	84.45	93.56	-9.10	-13.32	-4.89	2007	91.38	72.33	19.05	13.95	24.14
	Zimbabwe	2005	11.92	2015	32.46	10	20.54	2.05							2005	29.25	17.33	11.92	7.41	16.42

Table S4: Change in urban/rural inequities in the percentage of children under five whose birth is not registered in 67 countries (1999-2016)

Region	Country	% of children under five whose birth is not registered by iurban/rural residience and urban/rural difference						% of children under five whose birth is not registered by iurban/rural residience and urban/rural difference						Interval change in urban/rural difference (% points)					
		Wave 3 (2009-2012)						Wave 4 (2013-2015)											
		Year	Average (%)		Difference (% points)			Year	Average (%)		Difference (% points)								
			Rural	Urban	Estimate	95% CI			Rural	Urban	Estimate	95% CI		Wave 1 to 2	Wave 2 to 3	Wave 3 to 4	Wave 1 to 4	Wave 2 to 4	Wave 1 to 3
Sub-Saharan Africa	Angola							2015	86.40	67.14	19.26	15.89	22.62				4.82		
	Benin	2011	24.18	13.08	11.10	8.23	13.97								-3.46				
	Burkina Faso	2010	26.43	7.12	19.31	16.76	21.87								-8.17				
	Burundi	2010	25.89	13.44	12.45	8.46	16.44							6.05	10.39				16.44
	CAR	2010	48.38	21.65	26.73	22.47	30.99							11.94	-9.63				2.31
	Cameroon	2011	52.43	19.49	32.94	28.68	37.20	2014	46.92	15.80	31.12	26.55	35.69	32.14	4.90	-1.82	35.22	3.08	37.04
	Chad	2010	91.34	57.86	33.49	29.68	37.29	2014	93.61	64.37	29.23	25.60	32.87			-4.25	-5.83		-1.58
	Comoros	2012	13.55	10.48	3.07	-1.12	7.27												-1.31
	Congo DR	2010	70.82	76.14	-5.32	-10.74	0.10	2013	77.75	70.00	7.75	2.46	13.03	8.58	-7.46	13.07	14.18	5.61	1.11
	Cote dIvoire	2011	46.42	15.48	30.94	26.24	35.64							10.64	-7.78				2.85
	Gambia							2013	27.79	28.28	-0.48	-5.53	4.56	-4.46			-8.13	-3.68	
	Ghana	2011	44.81	28.03	16.79	12.31	21.27	2014	36.43	20.97	15.46	10.03	20.89		-9.64	-1.32		-10.96	
	Guinea Bissau							2014	82.46	65.55	16.91	12.74	21.09	35.00			31.89	-3.12	
	Kenya							2014	39.01	21.21	17.80	15.03	20.56					-1.78	
	Lesotho	2009	54.45	56.61	-2.15	-7.59	3.28	2014	59.87	46.13	13.74	7.46	20.01			15.89			
	Liberia							2013	79.91	70.85	9.06	4.24	13.88					6.61	
	Mali	2012	18.27	4.62	13.66	10.66	16.66								-16.43				
	Mauritania	2011	51.38	24.83	26.54	22.49	30.59								-6.24				
	Mozambique	2011	53.17	49.42	3.75	-0.38	7.88								-7.01				
	Namibia							2013	14.04	11.05	2.99	0.00	5.97					-20.18	
	Niger	2012	40.34	8.30	32.04	28.40	35.68							0.95	-13.91				-12.96
	Nigeria	2011	67.83	37.15	30.68	26.20	35.15	2013	81.44	50.18	31.25	27.66	34.85		2.98	0.58		3.56	
	Rwanda	2010	36.38	39.58	-3.20	-8.22	1.82	2014	43.90	44.57	-0.67	-6.25	4.91		1.22	2.53		3.74	
	S Tome & Principe							2014	7.03	3.64	3.39	0.46	6.32	-3.50			-2.69	0.81	
	Senegal	2010	34.08	10.68	23.40	19.86	26.94	2015	42.19	12.97	29.22	24.04	34.40			5.82	-3.42		-9.24
	Sierra Leone	2010	21.86	22.39	-0.52	-6.49	5.45	2013	24.24	20.40	3.84	-1.17	8.85	-14.21	-11.85	4.36	-21.70	-7.49	-26.06
	Swaziland	2010	53.54	38.49	15.04	9.38	20.70	2014	49.45	36.51	12.94	4.41	21.46	-12.90	5.04	-2.11	-9.96	2.93	-7.86
	Tanzania	2010	90.35	55.78	34.57	28.66	40.48	2015	82.31	49.08	33.23	28.55	37.91			-1.34			
	Togo	2010	29.39	6.68	22.71	18.94	26.47							8.31	-0.77				7.54
	Uganda	2011	71.32	61.98	9.34	3.37	15.31								6.39				
	Zambia							2013	93.32	79.65	13.67	10.39	16.96	28.15			22.78	-5.37	
	Zimbabwe	2010	57.31	34.81	22.50	17.98	27.02	2015	65.75	33.29	32.46	27.96	36.96		10.58	9.96		20.54	
